# Single‐Cell Transcriptomes of Immune Cells from Multiple Compartments Redefine the Ontology of Myeloid Subtypes Post‐Stroke

**DOI:** 10.1002/advs.202408722

**Published:** 2025-02-11

**Authors:** Mo Yang, Yixiang Li, Kaibin Shi, Xuezhu Wang, Xiangrong Liu, Xiang Huang, Fu‐Dong Shi, Shaojie Ma, Mingfeng Li, Yilong Wang

**Affiliations:** ^1^ Department of Neurology Beijing Tiantan Hospital Capital Medical University Beijing 100070 China; ^2^ Department of Pharmacology School of Basic Medicine Tongji Medical College Huazhong University of Science and Technology Wuhan 430030 China; ^3^ Laboratory for Clinical Medicine Capital Medical University Beijing 100069 China; ^4^ National Center for Neurological Disorders Beijing 100070 China; ^5^ Advanced Innovation Center for Human Brain Protection Capital Medical University Beijing 100069 China; ^6^ China National Clinical Research Center for Neurological Diseases Beijing 100070 China; ^7^ Beijing Laboratory of Oral Health Capital Medical University Beijing 100069 China; ^8^ Beijing Municipal Key Laboratory of Clinical Epidemiology Beijing 100069 China; ^9^ Chinese Institute for Brain Research Beijing 102206 China; ^10^ The Key Laboratory for Drug Target Researches and Pharmacodynamic Evaluation of Hubei Province Wuhan 430030 China; ^11^ Innovation center for Brain Medical Sciences Tongji Medical College Huazhong University of Science and Technology Wuhan 430030 China; ^12^ Chinese Institutes for Medical Research Beijing 100069 China; ^13^ Institute of Neuroscience CAS Center for Excellence in Brain Science and Intelligence Technology University of Chinese Academy of Sciences Chinese Academy of Sciences Shanghai 200031 China; ^14^ Key Laboratory of Computational Neuroscience and Brain‐Inspired Intelligence (Fudan University) Ministry of Education Shanghai 200433 China

**Keywords:** brain, immune cells, scRNA‐seq, skull bone marrow, stroke

## Abstract

The activation and infiltration of immune cells are hallmarks of ischemic stroke. However, the precise origins and the molecular alterations of these infiltrating cells post‐stroke remain poorly characterized. Here, a murine model of stroke (permanent middle cerebral artery occlusion [p‐MCAO]) is utilized to profile single‐cell transcriptomes of immune cells in the brain and their potential origins, including the calvarial bone marrow (CBM), femur bone marrow (FBM), and peripheral blood mononuclear cells (PBMCs). This analysis reveals transcriptomically distinct populations of cerebral myeloid cells and brain‐resident immune cells after stroke. These include a novel CD14^+^ neutrophil subpopulation that transcriptomically resembles CBM neutrophils. Moreover, the sequential activation of transcription factor regulatory networks in neutrophils during stroke progression is delineated, many of which are unique to the CD14^+^ population and underlie their acquisition of chemotaxis and granule release capacities. Two distinct origins of post‐stroke disease‐related immune cell subtypes are also identified: disease inflammatory macrophages, likely deriving from circulating monocytes in the skull, and transcriptionally immature disease‐associated microglia, possibly arising from pre‐existing homeostatic microglia. Together, a comprehensive molecular survey of post‐stroke immune responses is performed, encompassing both local and distant bone marrow sites and peripheral blood.

## Graphical abstract

Using single‐cell sequencing in a murine stroke model (p‐MCAO), three stroke‐specific subpopulations are identified: disease inflammatory macrophage (DIM), disease‐associated microglia (DAM), and CD14^+^ neutrophils (Neut_Cd14). Detailed analyses reveal that Neut_Cd14 and DIM subtypes are skull‐derived, whereas DAM likely originates from homeostatic microglia. This study highlights their gene expression, regulatory patterns, spatiotemporal distribution, and functional roles post‐stroke.

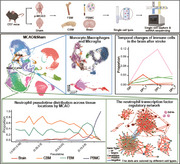

## 1 Introduction

Ischemic stroke is the second leading cause of preventable death and the third leading cause of long‐term disability. Survivors of stroke are challenged by stroke recurrence, long‐term sequelae, and cognitive decline, resulting in a huge socioeconomic burden worldwide.^[^ ^1^, ^2^, ^3^ ^]^ Innate and adaptive immune responses, especially those of acute phase are considered indispensable for stroke pathophysiology.^[^ ^4^ ^]^ Immediately after stroke, the release of damage‐associated molecular patterns (DAMPs) induces subsequent local and systemic immune responses.^[^ ^5^, ^6^ ^]^ Brain‐resident microglia senses and responds to stroke within minutes.^[^ ^7^ ^]^ Engrafted myeloid cells, especially neutrophils, are the primary immune cells infiltrating the core of infarction and penumbra from hours to days post‐stroke. Six hours after stroke, neutrophils can be detected in the meninges, with their number peaking at 24 h and decreasing from 48 to 72 h in the brain.^[^ ^8^ ^]^ Similar to neutrophils, the transcriptional shift in border‐associated macrophages is detected as early as 16 h after stroke and influences granulocyte transportation and vascular leakage.^[^ ^9^ ^]^ The transition of monocyte/macrophage subtypes in brain follows complex temporal patterns: Ly6c^hi^ monocytes infiltrate the infarcted area within 24 h of stroke and are gradually replaced by intermediate Ly6c^hi^/F4/80^hi^ cells by 3 days and, Ly6c^lo^/F4/80^hi^ macrophages by 6 days post‐stroke.^[^ ^10^, ^11^ ^]^ In general, peripheral monocytes/macrophages are attracted by cytokines and chemokines to brain 6 to 48 h after stroke.^[^ ^12^, ^13^ ^]^ Thus, several subtypes of myeloid cells, including neutrophils and early‐infiltrating monocyte/macrophage subtypes, dominate in cerebral immune responses 24 h after stroke. Serial gene expression and phenotypic changes in microglia, myeloid cells and lymphocytes further complicate post‐stroke immune “symphony”.^[^ ^14^, ^15^ ^]^ Nonetheless, the potential origins and gene expression profiles of immune cells in stroked brain during the acute phase, especially at the important timepoint 24 h after stroke, remain elusive.

Owing to the presence of the blood‐brain barrier (BBB) and its developmental characteristics, the brain was once considered an “immune‐privileged” organ. Although the cell types that colonize the central nervous system (CNS) respond quickly to brain injuries, some viewpoints hold that most myeloid cells and lymphocytes emerge in brain secondary to cerebral injuries and infiltrate the damaged BBB, choroid plexus, or recently reported microchannels connecting the cranial bone marrow (CBM) and dura,^[^ ^16^, ^17^, ^18^, ^19^ ^]^ and then exit the dura and brain via arachnoid cuff exit bridge.^[^ ^20^ ^]^ Interestingly, Nahrendorf et al. found that the skull bone marrow contributed more neutrophils and monocytes to the brain than the remote femur after a stroke or in other pathological conditions.^[^ ^16^ ^]^ The bidirectional transport of pathogens or immune cells via abovementioned microchannels may illicit immune responses in the CBM,^[^ ^21^ ^]^ highlighting the importance of the skull in immune surveillance of the brain. However, the immune response alterations of the brain, skull, and tissues from which peripheral myeloid cells originate, as well as differences among the above tissues in terms of homeostatic and post‐stroke states, are not comprehensively understood. In the present study, we provide a transcriptomic and ontological landscape of local and migrated immune cells in the ischemic brain, CBM, femur, and peripheral blood post ischemic stroke.

Among the previous publications that touched on the gene expression features of post‐stroke CBM, two were in the context of ischemic stroke. Kolabas et al. identified the transcriptomic features of mouse CBM, vertebrae, brain, and multiple peripheral bone marrow sites 3 days after the induction of transient middle cerebral artery occlusion (t‐MCAO), and they focused almost exclusively on neutrophils from the subacute phase of stroke.^[^ ^22^ ^]^ Another recent study by Xu et al. also utilized the t‐MCAO model to compare gene expression differences in the ipsilateral and contralateral cranial bone flaps 15 min after reperfusion, and identified osteopontin as an important signal promoting lateralized CBM inflammation.^[^ ^23^ ^]^ However, although the t‐MCAO model is associated with a smaller ischemic volume and higher survival rate than the permanent middle cerebral artery occlusion (p‐MCAO) model,^[^ ^24^ ^]^ it is representative of the relatively small proportion of stroke patients who achieve early recanalization. Notably, ≈90% stroke patients cannot realize effective recanalization owing to reocclusion, a limited therapeutic time window, contraindications to reperfusion therapy,^[^ ^25^, ^26^ ^]^ or treatment unavailability in certain regions or countries.^[^ ^27^ ^]^ Thus, the t‐MCAO model, particularly when promptly reperfused, is considered unsuitable for translational research.^[^ ^28^ ^]^ In addition, the immune responses in the two experimental models vary. p‐MCAO rodents have a higher number of neutrophils’ transmigrating into the hemisphere contralateral to the stroke and diverse polarization and anti‐inflammatory phenotypes of brain‐infiltrating macrophages and monocytes compared to t‐MCAO mice, differences which underlie heterogenous transcriptomic reprogramming after stroke.^[^ ^29^ ^]^ Moreover, previous studies investigated post‐stroke immune responses in either the hyperacute^[^ ^23^ ^]^ or subacute^[^ ^22^ ^]^ phases of stroke but not in the acute phase when myeloid cells peak and dominate inflammation. Moreover, most previous studies have overlooked the uncertain ontology or interconnections of myeloid cells from different potential origins, such as cranial or peripheral bone marrow, and stressed the transcriptomic features of a single tissue.^[^ ^22^, ^23^, ^30^, ^31^, ^32^, ^33^ ^]^ Thus, despite recent studies investigating the gene expression features of the CBM,^[^ ^22^, ^23^ ^]^ the transcriptomic and ontological landscape of local and migrated immune cells in the ischemic brain, CBM, femur, and peripheral blood remains uncharacterized.

## 2 Results

### 2.1 Transcriptomic Profiling of Immune Cells from Brain and Peripheral Tissues Post‐Stroke

**Figure 1:**
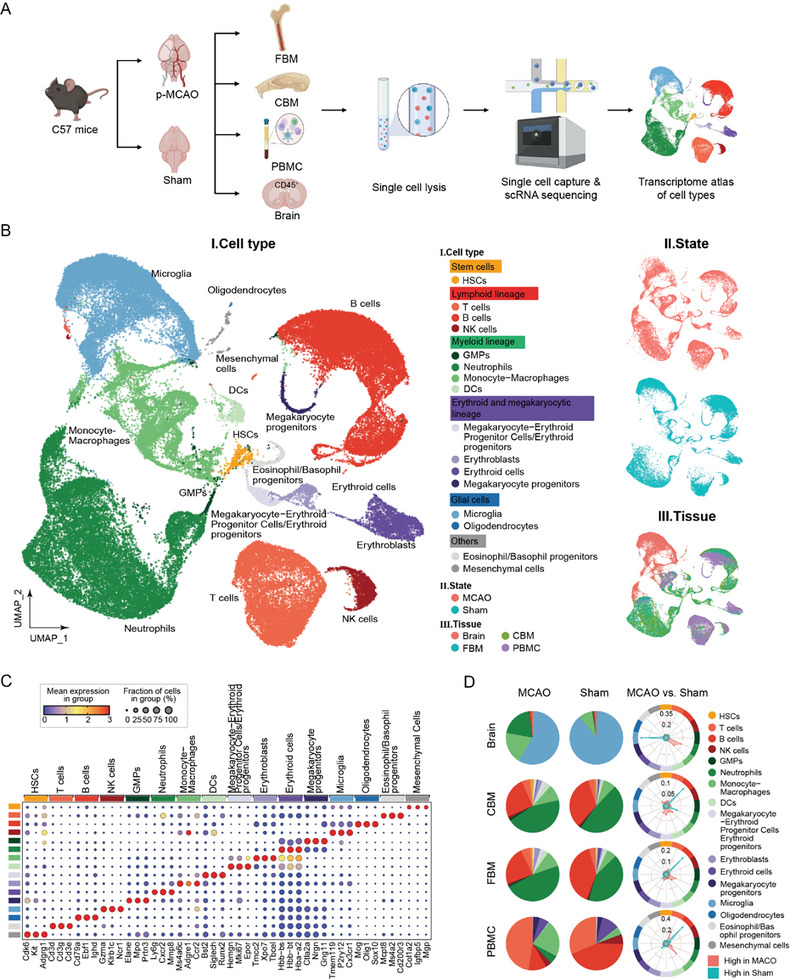
A molecular landscape of immune cells in stroked and sham mice. A) Scheme of experimental design. Single cells were sorted from various tissues in the p‐MCAO and Sham groups for scRNA‐seq analysis. Parts of Figure 1A were created in BioRender. Yang, M. (2025) https://BioRender.com/r05m679. B) UMAP plot showing clusters of cells identified and annotated in each tissue after 24 h in the p‐MCAO‐induced and Sham groups. C) Dotplot depicting the expression of marker genes in each cell type. The color of the dots indicates the average gene expression level in that cell type, and the size indicates the percentage of cells expressing the gene in that cell type. D) Pie charts showing the percentage of each cell type in both the p‐MCAO and Sham groups for all four tissues, as well as radar plots displaying the absolute difference in the change of cell type proportions between the p‐MCAO and Sham groups in each tissue.

To better decode the gene expression alterations of immune cells in the brain post‐stroke and delineate their precise tissue origins, we conducted single‐cell RNA sequencing (scRNA‐seq) of sorted CD45^+^ cells from brain and potential distant origins for CNS‐infiltrated immune cells, including CBM, femur bone marrow (FBM), and peripheral blood mononuclear cells (PBMCs). Notably, we did not investigate the transcriptomic features of other immune organs, such as the spleen or lymph nodes, which might play important roles in systemic inflammation but were not major sources of cerebral immune cells. Both p‐MCAO and sham mice were analyzed (**Figure** **1**A). We chose the time point 24 h post‐stroke to harvest tissues.

After stringent quality control, we retained 21 841 cells from the brain, 19 852 cells from the calvaria, 18 469 cells from the femur, and 22 885 cells from PBMCs (Figure S1, Supporting Information). Unsupervised clustering identified 16 cell types marked by canonical markers, showing a clear separation by cell type and tissue origin. These cell types formed multiple continuums on the uniform manifold approximation and projection (UMAP), representing lineages starting from stem cells and giving rise to lymphoid, myeloid, erythroid, and megakaryocytic cells, and others (Figure 1B,C; Figure S1B, Supporting Information). While the UMAP plot demonstrated limited separation between the immune cells from the p‐MCAO and sham groups, the relative cell density varied significantly between the two groups, suggesting possible changes in cell proportions post‐stroke (Figure 1D).

Dissecting tissue‐specific constituents and proportions of different immune cell subsets may shed light on their functions and origins. Therefore, we compared the relative proportions of different cell types in each tissue between the p‐MCAO and sham groups (Figure 1D). In the brain, CD45^+^ microglia decreased from 88.6% to 57.2% 24 h post‐stroke. In contrast, the neutrophil population increased dramatically (from 0.7% to 19.6%), accompanied by a moderate proportion increase in monocytes/macrophages (from 8.1% to 19.2%) (Figure 1D; Figure S1C and Table S1, Supporting Information). These findings are consistent with previous reports that myeloid cells, such as neutrophils and monocytes/macrophages, are among the earliest cells to infiltrate the ischemic brain.^[^ ^7^, ^15^ ^]^

In the PBMC dataset, eight and nine cell types were utilized in the p‐MCAO and sham groups, respectively, for conditional comparisons. Erythroid cells were excluded to prevent potential sample variations caused by insufficient red blood cell lysis. Since neutrophils constitute a large proportion of post‐stroke immune cells in the peripheral blood, we performed density gradient centrifugation to discard some mature neutrophils to enrich for other myeloid or lymphoid cell types while preserving representative neutrophil subsets. After ischemic stroke, the percentage of B cells decreased distinctly from 43.2% to 10.9%, whereas myeloid lineage cells were increased in PBMCs after stroke, indicating more active mobilization of myeloid cells from the remote bone marrow to the peripheral blood (Figure 1D; Figure S1C and Table S1, Supporting Information).

Cells from CBM and FBM tended to cluster together on the UMAP, indicating similar transcriptomic features of the two bone marrow sites (Figure 1B). Our analyses revealed an increase in the proportion of hematopoietic stem cells (HSCs), T cells, natural killer cells, granulocyte‐monocyte progenitors (GMPs), monocytes/macrophages, dendritic cells (DCs), eosinophil/basophil progenitor cells, and erythroblasts in both the ischemic CBM and FBM groups compared to those in the sham group (Figure 1D), indicating their proliferation and activation in both CBM and FBM after ischemic injury. However, the proportion of neutrophils changed differently in CBM and FBM post‐stroke, decreasing from 47.9% to 44.3% in CBM and becoming slightly more abundant (41.7% to 47.7%) in FBM. In line with our finding, another study reported similar decrease of neutrophil cell count in CBM, but not in FBM, after ischemic stroke using flow cytometry analysis.^[^ ^16^ ^]^ Building on these findings, we propose that there may be homogeneity and tissue‐specific divergence between the CBM and FBM neutrophils in response to stroke.

### 2.2 Myeloid Cells, Especially Neutrophils, Exhibit Tissue‐Specific Transcriptomic Features

**Figure 2:**
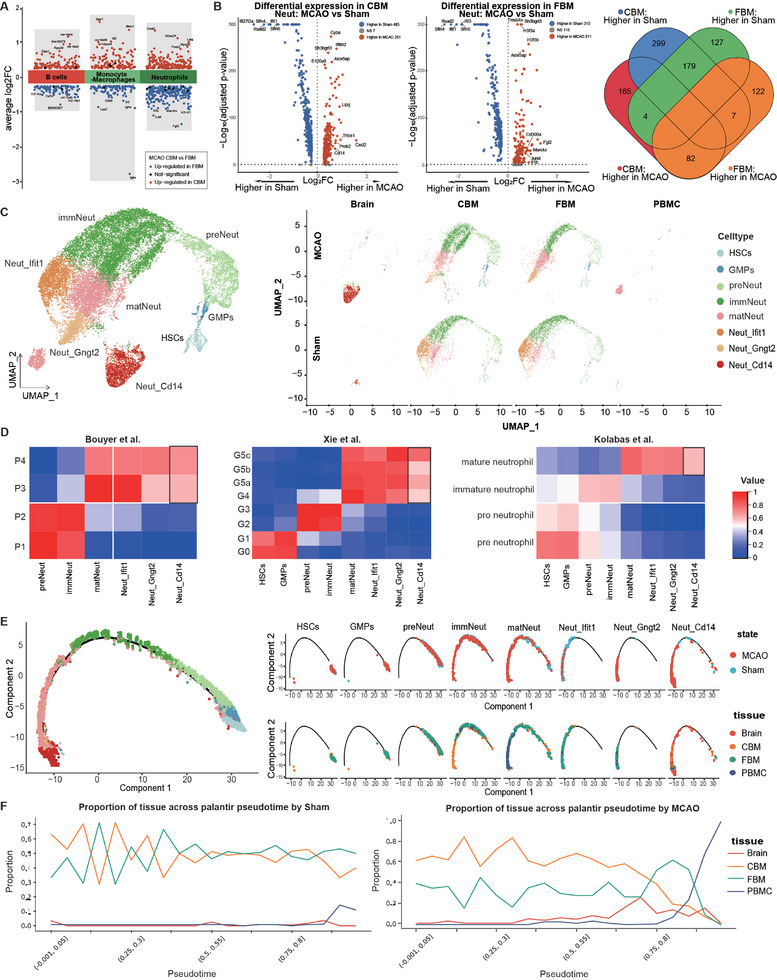
Neutrophil gene expression and differentiation in stroke. A) Cell types exhibiting greatest tissue‐specific transcriptomic variance comparing CBM and FBM in the stroke mice. B) Volcano plots showing differentially expressed genes in neutrophils in CBM and FBM between p‐MCAO and sham groups, respectively. Red represents up‐regulation in the p‐MCAO group, blue represents up‐regulation in the sham group, and p_val_adj < 0.05 is considered significantly differentially expressed. Venn plots show the count of differently expressed genes in the two bone marrow tissues. C) UMAP plots of HSC, GMP, and neutrophil subtypes clustered (left), and UMAP plots split by state and tissue (right). D) The data in this article were selected for correlation comparison with the data of Bouyer et al. (left), Xie et al. (middle), and Kolabas et al. (right), and the color indicates the correlation of each subcluster. E) Differentiation trajectories of neutrophil subtypes, colored by cell type (left). Position of different subtypes on the differentiation trajectory (right), coloring cells by state in the top panel and tissue origin in the bottom panel. F) Proportion of tissue across palantir pseudotime by state. Division of pseudotime into 20 intervals, color‐coded by tissue.

To delineate tissue‐specific immune responses after stroke, we first compared the CBM and FBM, two important contributors of immune cells in the brain. Interestingly, among the major immune cell types analyzed, neutrophils, followed by monocytes/macrophages, demonstrated the most evident transcriptomic divergence, contrasting the two bone marrow sites post‐stroke (**Figure** **2**A; Figure S2B, Supporting Information). In addition, a small group of CBM neutrophils was located close to the cerebral neutrophils in our 3D UMAP analysis, whereas FBM neutrophils did not present a similar spatial layout (Figure S2A, Supporting Information). We inferred that the CBM produces and transports more neutrophils to the brain than the FBM and that there may be underlying transcriptomic differences between the CBM and remote FBM, distinguishing their tissue heterogeneity. Thus, we first investigated the gene expression characteristics of neutrophils, which exhibit significant tissue variances and are the earliest recruited myeloid cells in the ischemic brain.

After stroke, CBM neutrophils preferentially increased the expression levels of *Cxcl2*, *Prok2*, *Ngp*, *Thbs1*, and *Cd177*. *Cxcl2* encodes the chemokine CXCL2, which is known for its role in regulating the circadian rhythm of neutrophil aging and egression from blood vessels by binding to CXCR2.^[^ ^34^ ^]^ Similarly, *Prok2/BV8*, a gene sharply up‐regulated in neutrophils to modulate cell migration via the ERK and PI3K pathways in response to granulocyte‐colony stimulating factor,^[^ ^35^ ^]^ was highly expressed in the CBM. As previously reported, the expression of *Cd177* in the bone marrow and blood neutrophils was downregulated after stroke as neutrophils differentiate and mature.^[^ ^36^ ^]^ Conversely, neutrophils generated from FBM were skewed to higher expression of *Fgl2, Ccl6, IL1b, Marcks*, and *H2‐K1* post‐stroke, which are related to inflammation resolution,^[^ ^37^ ^]^ pyroptosis and extracellular trap formation.^[^ ^38^ ^]^ Gene Ontology (GO) enrichment analysis of tissue‐specific differentially expressed genes revealed that CBM neutrophils were notably linked to functions such as energy metabolism and myeloid cell migration, while their FBM counterparts were closely associated with cell cytotoxicity, cellular aging, and immune regulation. Additionally, the divergence in gene expression extended beyond neutrophils, encompassing various cell types (Figure S2C, Supporting Information). Together, the heterogeneous gene expression signatures observed across these two marrow sites suggest heightened metabolic activity and vigorous neutrophil migration in the CBM, in contrast to the relatively more mature immune functions observed in FBM neutrophils, indicating the tissue‐specific gene expression profiles of neutrophils after stroke.

Next, we included the sham group to explore whether the transcriptomic shifts of neutrophils in response to stroke were divergent between the CBM and FBM. We examined genes that were upregulated and downregulated in response to stroke and compared these differentially expressed genes between CBM and FBM (Figure 2B). In stroke‐challenged mice, 165 genes were specifically upregulated and 299 genes were specifically down‐regulated in the CBM, whereas fewer genes were upregulated (*n* = 122) or downregulated (*n* = 127) in the FBM. Intriguingly, fewer genes were shared between the CBM and FBM in terms of upregulation (*n* = 82) or downregulation (*n* = 179), which was much lower than the number of tissue‐specific genes. These results also suggest tissue‐specific responses of neutrophils to stroke.

### 2.3 Transcriptomic Characterization of Neutrophil Subpopulations

**Figure 3:**
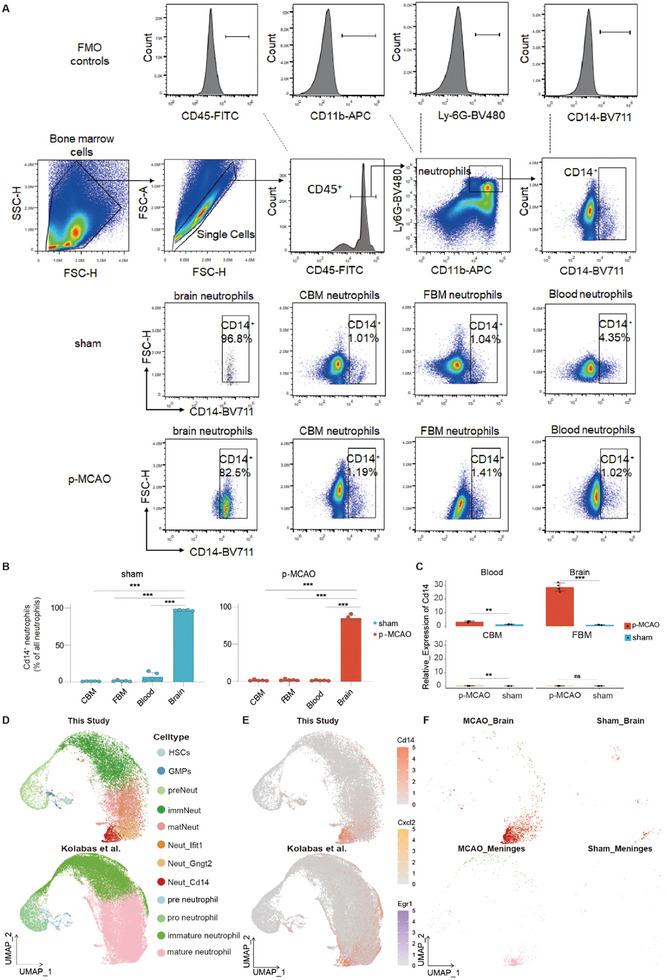
Identification of Neut_Cd14 subtype and its correlation with other datasets. A) FACS staining strategy and detection of CD14^+^ neutrophils (*n* = 5 per group). B) Percentage of CD14^+^ neutrophils in sham and MCAO‐induced CBM, TBM, blood, and brain neutrophils (*n* = 5 per group). Statistical significance was determined by one‐way ANOVA followed by Tukey's post‐hoc test. C) Relative expression of Cd14 in neutrophils from CBM, TBM, blood and cerebral origins (*n* = 4 per group). Statistical significance was determined by paired t test. D) The CCA algorithm was applied to integrate the neutrophil lineage data from this study with the neutrophil data from Kolabas et al.^[^ ^22^ ^]^ The UMAP data from this study is shown in the upper layer with the defined cell cluster coloring, and the data from Kolabas et al.^[^ ^22^ ^]^ is shown in the lower layer with their defined cell cluster coloring. E) The cells expressing the three DEGs of the Neut_Cd14 subcluster are shown in different colors on the UMAP after data integration, while those not expressing these genes are colored gray. F) The data of (D) in the brain from this article and the meninges data from (D) in Kolabas et al.^[^ ^22^ ^]^ were separated by states in the UMAP plot. Our data is in the upper layer, and that of Kolabas et al.^[^ ^22^ ^]^ is in the lower layer. Data presented as mean ± SEM, with *p < 0.05, **p < 0.01, ***p < 0.001.

Neutrophils exhibit complex phenotypes and gene expression features under different disease conditions and spatial locations. Thus, we focused on neutrophil subpopulation annotation to investigate tissue‐specific features and developmental trajectories. We included HSCs and GMPs as natural roots to complete the neutrophil differentiation and maturation process. Leveraging unsupervised clustering and canonical markers, we identified the subtype Neut_Cd14 (*Cd14* ^+^, *Cxcl2* ^+^ *, Egr1* ^+^), which has not been characterized previously, and five known subtypes representing different maturation stages of neutrophils including preNeut (*Camp* ^+^, *Ngp* ^+^, *Ltf* ^+^, *Fcnb* ^+^, *Tuba1b* ^+^, *Chil3* ^+^), immNeut (high levels of *Camp*, *Ngp*, *Ltf*, and intermediate levels of *Cxcr2*, *Mmp8, Retnlg*), matNeut (*Cxcr2* ^+^, *Mmp8* ^+^, *Retnlg* ^+^), Neut_Ifit1 (*Ifit1* ^+^ *, Ifit3* ^+^ *, Rsad2* ^+^ *, Isg15* ^+^) and Neut_Gngt2 (*Gngt2* ^+^, *Gm2a* ^+^, and *Fgl2* ^+^) (**Figure** **3**C; Figure S3, Supporting Information).^[^ ^39^, ^40^, ^41^ ^]^ To delineate the identity of these defined neutrophil subtypes, we conducted comprehensive integration with external neutrophil datasets from three independent publications.^[^ ^22^, ^39^, ^42^ ^]^ We observed a strong correspondence with prior studies in hierarchical correlation analysis, even reproducibly detecting close homologous subtypes across the three datasets (Figure 2D). Nonetheless, the Canonical Correlation Analysis (CCA) integration with each of the three datasets revealed that the homologous subtypes assembled closely in UMAPs (Figure S4, Supporting Information). Notably, Neut_Cd14 consistently exhibited weaker correlations with the most mature neutrophil subtypes compared to the correlation values observed between homologous subtypes. Analysis of the tissue origin revealed that the Neut_Cd14 subtype was overwhelmingly located in the ischemic brain (Figure 2C). Taken together, we hypothesize that the previously uncharacterized Neut_Cd14 might play a key role in stroke immunity.

Monocle was used to construct the cell trajectory and examine the developmental stages of the five neutrophil subtypes.^[^ ^43^ ^]^ Notably, due to the predominantly multinuclear nature of mature neutrophils and their tendency to be discarded during the isolation of PBMCs, PBMC neutrophils were not discussed. Our analysis revealed a single continuous branch for neutrophil maturation, with the continuum ending predominantly with Neut_Gngt2 and Neut_Cd14 (p‐MCAO) or Neut_Ifit1 (sham) subpopulations (Figure 2E), suggesting relatively more mature developmental roles for these subtypes.

GO enrichment analysis of the subtype markers revealed that preNeut, immNeut, and matNeut neutrophils were enriched for genes relating anabolic or metabolic activities, whereas Neut_Ifit1, Neut_Gngt2, and Neut_Cd14 neutrophils were enriched for genes associated with immune functions, such as combatting infections or regulating innate immunity (Figure S3E, Supporting Information). In particular, Neut_Gngt2 and Neut_Cd14 emerged after stroke and were related to active cell proliferation, differentiation, and chemokine responses. Collectively, preNeut, immNeut, and matNeut neutrophils correlated with relatively naïve gene signatures, whereas Neut_Ifit1presented quiescent mature neutrophil features, preparing for potential infectious threats, and Neut_Gngt2 and Neut_Cd14 acted as activated/mobilized mature neutrophils post‐stroke. Thus, our analyses revealed the gene expression features and functions related to different neutrophil subsets.

Although located close together on the UMAP, differential gene expression analysis and similarity profiling further substantiated the distinct transcriptomic identity of the Neut_Cd14 and Neut_Gngt2 subsets. These analyses revealed differences in the number of specific genes expressed by each subset and a limited similarity between the two populations (Figure S5B, Supporting Information), suggesting that they are separate and specialized subgroups. Additionally, GO enrichment analysis tools, such as msigdbr and clusterProfiler, revealed shared and distinct functional pathways associating Neut_Cd14 and Neut_Gngt2 (Figure S5, Supporting Information). While both subpopulations were involved in common immune responses, such as cytokine production and leukocyte migration, Neut_Cd14 was primarily associated with processes related to apoptosis, neuronal death, and the regulation of immune responses. In contrast, Neut_Gngt2 was associated with functions related to actin cytoskeleton dynamics, facilitating immune cell migration by regulating actin polymerization, depolymerization, and reorganization.

### 2.4 Cerebral Neutrophils Emerging After Stroke May Originate from Skull

Flow cytometry was used to validate the presence of Neut_Cd14 in murine brain after stroke. We isolated Neut_Cd14 based on the surface expression of CD14 (Figure 3A). Our results showed that CD14^+^ neutrophils constituted a remarkable proportion of brain neutrophils (>80%), but only a minimal proportion of peripheral blood, calvaria, or femur neutrophils (<5%) (Figure 3B). We also evaluated the relative expression of *Cd14* in neutrophils from the brain, CBM, FBM, and blood, and confirmed high expression of *Cd14* in the ischemic brain, with the post‐stroke increase in Cd14 expression being most significant in brain (Figure 3C). Therefore, we indeed identified the tissue‐specific “tropism” of Neut_Cd14, which predominantly emerged in brain. UMAP and cell lineage trajectory analyses showed that Neut_Cd14 was associated with upstream calvarial neutrophil subsets. These results suggest that brain CD14^+^ neutrophils may be derived from the calvaria, but not from the peripheral bone marrow (Figure 2C,E).

Next, we performed a pseudotime analysis of our neutrophil subpopulations and upstream HSCs and GMPs using the Palantir and scVelo methods (Figure S6, Supporting Information). The results indicated that the cell trajectory of neutrophils was comparable between the CBM and FBM in the sham states. However, a disruption of the balance between CBM and FBM neutrophils was observed in the p‐MCAO model (Figure 2F; Figure S6, Supporting Information). Pseudotime trajectory analysis revealed that after MCAO, the proportion of CBM neutrophils was higher than that of FBM at each early cell stage prior to neutrophils’ migration into the brain. In contrast, the proportions of CBM and FBM neutrophils were reversed following cerebral neutrophil migration. The results thus indicate earlier differentiation and mobilization of neutrophils and their progenitor cells in the CBM than in the FBM after stroke, before their potential migration into the brain and later maturation process.

To confirm this finding further, we revisited two external datasets. First, we analyzed the cranial neutrophil data from Xu et al., who studied the skull transcriptome on the contralateral and ipsilateral sides of stroke.^[^ ^23^ ^]^ We defined Neut_Cd14 in the pooled dataset based on the co‐expression of its three marker genes (Neut_Cd14_DEG>0_cells). Our results indicated that immune responses were initiated in the affected cranial bone as early as 1 h after ischemia, with the presence of Neut_Cd14_DEG>0_cells detected in the ipsilateral skull, but not in the contralateral skull. Furthermore, transcription factor analysis of the Neut_Cd14 subpopulation revealed that, within 1 h of stroke onset, specific transcription factors related to Neut_Cd14 differentiation were expressed in the affected skull (Figure S7D, Supporting Information). These findings support the hypothesis that the cerebral Neut_Cd14 subpopulation originates from the skull, and the process may be initiated quickly on the ipsilateral side of ischemia (Figure S7, Supporting Information). Next, we replotted the UMAP incorporating the Kolabas’ dataset,^[^ ^22^ ^]^ and found that Neut_Cd14 neutrophils were located at the juncture of their mature neutrophils, partially overlapping with them as well. Analysis of the representative marker genes of Neut_Cd14 neutrophils, such as *Cd14*, *Cxcl2*, and *Egr1*, revealed detectable co‐expression in meningeal neutrophils from the Kolabas’ dataset and their manifold proximity to Neut_Cd14 neutrophils (Figure 3D–F; Figure S8, Supporting Information). While further lineage tracing experiments may provide additional insights, the results indicate that the skull may reserve and transport neutrophils, to the meninges and then to the brain parenchyma, with the stroke‐ipsilateral crania responds the quickest.

### 2.5 Transcriptional Regulons Underlie Neutrophil Proliferation and Maturation

**Figure 4:**
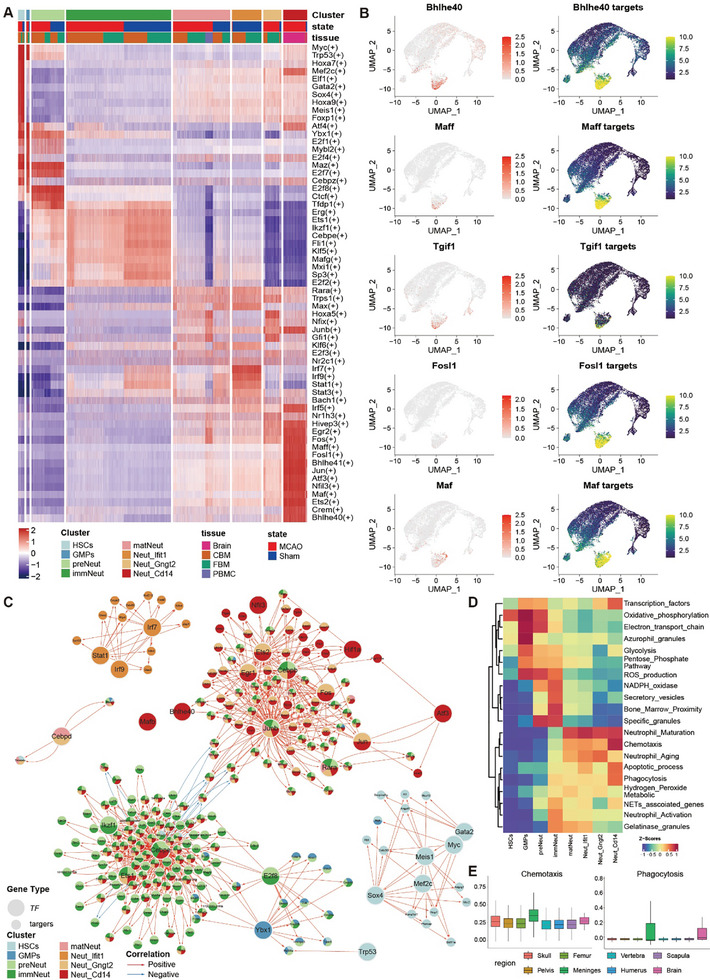
Neutrophil transcriptional regulatory network and cellular function. A) Complex heatmap plot of the top 10 regulatory factors in each cluster. B) UMAP plot showing the distribution of highly active TFs and their target genes in the Neut_Cd14 subcluster. TFs with an expression level > 2.5 are denoted by 2.5, and their expression level is represented by a gradient of red color. Target genes are shown with the 95% quantile as the upper limit of expression and the 5% quantile as the lower limit of expression, and the Viridis palette is used to indicate the different levels of expression. C) Predicted TF regulatory network with nodes colored by neutrophil subtype. The direction of the arrow indicates the targeting relationship, and the color shade indicates the level of correlation, with red indicating a positive correlation and blue indicating a negative correlation. D) Heatmap of cellular function scores. E) Box plot of functional scores of mature neutrophils in different regions from Kolabas et al.^[^ ^22^ ^]^ data.

Next, we investigated the transcriptional mechanisms that regulated post‐stroke neutrophil proliferation and maturation. Here, we utilized pySCENIC to integrate gene co‐expression and transcription factor (TF) motif enrichment to identify regulons, each consisting of a TF and its target genes.^[^ ^44^ ^]^ A total of 113 TF regulons were identified, all of which exhibited specificity for a subset of neutrophil subtypes (**Figure** **4**A; Figure S9, Supporting Information). For instance, Neut_Ifit1neutrophils exhibited increased expression of *Irf7*, *Irf9*, *Stat1*, and *Stat3*, which have previously been recognized for their roles in neutrophil antiviral immunity through the modulation of type I IFNs or IFN‐stimulated genes, suggesting their potential involvement in driving the specific maturation of neutrophils into the steady‐state Neut_Ifit1 subtype (Figure 4A).^[^ ^45^, ^46^ ^]^ Similarly, the increased expressions of TFs such as *Fos*, *Fosl1*, *Bhlhe40*, *Bhlhe41*, *Jun*, *Atf3*, *Nfil3*, *Maff*, and *Maf* were notably enriched in Neut_Cd14 neutrophils (Figure 4A). It is known that, *Fos, Fosl1, and Jun* interact with AP‐1‐binding sites, playing crucial roles in cell proliferation, differentiation, and neutrophil recruitment,^[^ ^47^, ^48^ ^]^ whereas *Atf3* regulates murine neutrophil migration,^[^ ^49^ ^]^ *Bhlhe40* and *Bhlhe41* modulate the circadian clock, polarization, and infiltration of neutrophils downstream of endoplasmic reticulum stress and hypoxia in tumors.^[^ ^50^, ^51^ ^]^ Nonetheless, the analysis of their gene targets, such as those associated with *Bhlhe40, Maff, Tgif1, Fosl1*, and *Maf*, consistently revealed increased expression levels in different neutrophil subtypes as they differentiated into the Neut_Cd14 subtype (Figure 4B). Reactome pathway analysis also indicated functional enrichment of these regulons, such as in cell chemotaxis and regulation of the innate immune responses, emphasizing their roles in delineating neutrophil states (Figure S10, Supporting Information). Together, these TF regulatory networks underlie the proliferation, differentiation, and maturation of neutrophils, including lineage specification and commitment of Neut_Cd14.

Next, we dissected the interplay between these TF regulons by identifying cross‐linked regulons, which resulted in 27 TFs and 222 putative target genes (Figure 4C). These results highlight the sequential employment of inter‐connected TF regulons that underlie the differentiation of stem cells into immature, and eventually mature neutrophil subtypes. Notably, *Myc, Gata2, Meis1*, and *Sox4*, which are important for the generation and survival of HSCs, the activation of long‐term hematopoietic stem cells, and oxidant defense, were specifically enriched in the HSC cluster.^[^ ^52^, ^53^, ^54^ ^]^ Additionally, *Cebpe, E2f8, Ikzf1*, and *Ets1*, known to pattern neutrophil generation from GMPs,^[^ ^55^ ^]^ survival and migration of neutrophil progenitors,^[^ ^56^ ^]^ cell cycle progression from G1 to S phase,^[^ ^57^ ^]^ and granulocytic maturation,^[^ ^58^ ^]^ respectively, were enriched in preNeut or immNeut neutrophils. Along with *Fos* and *Jun*, *Cebpb*, *Ets2*, *Egr1*, and *Junb* were enriched in Neut_Gngt2 and Neut_Cd14, particularly after stroke. *Cebpb* is associated with neutrophil emergency granulopoiesis in murine myocardial infarction;^[^ ^59^ ^]^ *Ets2* modulates genes associated with development and apoptosis, and its expression is upregulated in blood polymorphonuclear leukocytes 24 h after stroke.^[^ ^60^ ^]^ Expression of *Egr1* is upregulated in the ischemic hemisphere with its deletion associated with larger infarction volume and worse neurological scores.^[^ ^61^ ^]^ All of which indicating a functional switch of neutrophils into mature states. In contrast, *Mafb*, *Nfil3*, and *Hif1a* were specifically expressed only in Neut_Cd14, which might be associated with degranulation (*Nfil3*) or hypoxic response (*Hif1a*). Collectively, the serial regulation of these TFs and their target genes, as well as the interplay of TF networks may modulate the differentiation of neutrophil subtypes in sham or stroke‐induced mice.

Finally, we examined the cellular functions of each neutrophil subtype (Figure 4D). Overall, the anabolic function scores including oxidative phosphorylation, glycolysis, and NADPH oxidase, were higher in HSCs, GMPs, and immature neutrophils. In relatively mature neutrophil subclusters, biological functions such as phagocytosis, neutrophil chemotaxis and apoptosis were high, especially in the Neut_Cd14 subset (Figure 4D). We further evaluated functional scores representative of mature Neut_Cd14 in different tissues from the study by Kolabas et al.,^[^ ^22^ ^]^ and found that chemotaxis and phagocytic functions were most prominent in meningeal and cerebral neutrophils, supporting the hypothesis that mature CD14^+^neutrophils might engraft from the CBM to the meninges, and finally into the brain (Figure 4E). In addition, the upregulation of different regulons in coherent neutrophil subpopulations may accelerate neutrophils’ maturation and engraftment in the brain.

### 2.6 Transcriptomic Characterization of Monocytes/macrophages and Microglia

**Figure 5:**
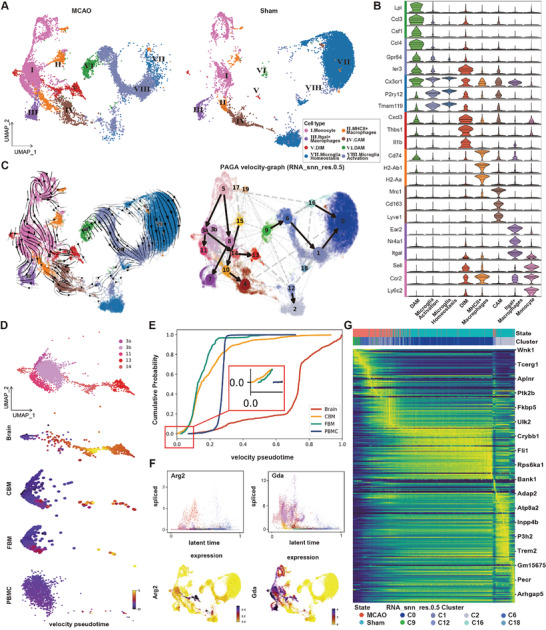
Reclassification and differentiation characteristics of monocyte‐macrophages and microglia. A) UMAP plots after re‐clustering analysis of monocytes/macrophages and microglia clusters are shown by state. B) Violin plots show the expression of marker genes in each cell type in A. C) RNA velocity stream plots (left). Cell cluster PAGA plots at 0.5 resolution (right). D) A scatter plot based on cell tissue state shows the distribution of cells associated with DIM differentiation pathways in different tissues, color‐coded for velocity pseudotime. E) Cumulative probability distribution of velocity pseudo‐times for different tissue states. F) Scatter plots depicting the relationship between gene expression and latent time of the pertinent chemotactic genes *Arg2* and *Gda* (left), and UMAP plots corresponding to their expression levels (right). G) Complex heatmap plot of microglia maturation and differentiation pathways. demonstrating alterations in gene expression patterns between different cell states and clusters with latent time.

The monocytes/macrophages population is another important branch of myeloid cell types in stroke immunity and is the second most tissue‐variant cell type comparing CBM and FBM (Figure 2A). In principle, post‐stroke brain macrophages comprise monocyte‐derived macrophages supplemented from either the CBM or damaged BBB as well as brain‐resident macrophages, including meningeal, choroid plexus, and perivascular macrophages.^[^ ^62^, ^63^ ^]^ We extracted monocytes/macrophages along with microglia to conduct refined clustering analysis (**Figure** **5**A; Figure S11, Supporting Information). The inclusion of microglia is due to its consistent classification as another type of “tissue macrophages”, sharing similar ontogeny and phagocytic capacity with most border‐associated macrophages.^[^ ^63^, ^64^ ^]^

Based on the outcome of unsupervised clustering and enriched canonical gene markers, we eventually defined five monocytes/macrophages subtypes and three microglial subtypes (Figure 5A,B).^[^ ^65^, ^66^, ^67^, ^68^ ^]^ Regarding the monocytes/macrophages subtypes: the monocytes consisted of relatively immature cells expressing *Ly6c2*, *Ccr2*, *Sell*, and *Itgal*; the Itgal^+^ macrophages expressed *Itgal* (encodes CD11a), *Ccr2*, *Nr4a1*, *Ear2*; the disease inflammatory macrophage (DIM) population was characterized by high expression of *Il1b*, *Thbs1*, *Cxcl3* and *Ier3*; the CNS associated macrophages (CAM) highly expressed *Lyve1*, *Cd163*, and *Mrc1*; and the MHCII^+^ expressing macrophages featured *Ccr2*, *H2‐Aa*, *H2‐Ab1* (encodes MHCII), and *Cd74*. Notably, DIMs were present mainly in stroke‐induced brains (Figure S11, Supporting Information).

As for the three microglia subtypes: the disease‐associated microglia (DAM) were characterized by the expression of *Cx3cr1*, *Ier3*, *Gpr84*, *Ccl4*, *Csf1*, *Ccl3*, and *Lpl*. The microglia homeostasis subtype exhibited high expression of *Tmem119*, *P2ry12*, and *Cx3cr1*; and the microglia activation subtype also showed high expression of *Tmem119*, *P2ry12*, and *Cx3cr1*, but lower expression levels of gene markers specific to DAM, such as *Ier3* and *Gpr84* (Figure 5B). Notably, all macrophage and microglia subtypes, but not monocytes, highly expressed *Cx3cr1*, coinciding with previous findings that monocytes enter the brain and transform into CX3CR1^+^ amoeboid microglial cells or “M2”‐like macrophages. (Figure 5B).^[^ ^69^, ^70^ ^]^ Hence, *Cx3cr1* was discerned as a ticket for myeloid cells to roll and adhere alongside vessels and engraft into the inflamed brain.^[^ ^71^ ^]^

### 2.7 Post‐Stroke DIMs and DAM Showed Heterogeneous Origins

We further attempted to discern the potential origins and associated functions for monocytes/macrophages and microglia. First, we analyzed RNA velocity and predicted the differentiation trajectories (see Method). To obtain more intermediate snapshots, the eight cell subtypes were further subdivided into 21 clusters (termed C0‐C19). Notably, C3 monocytes were further subdivided into C3a and C3b, as C3a was predominantly located in the CBM (39.0%), FBM (40.9%), and brain (20.1%), whereas C3b was exclusively found in PBMCs (Figure 5C). Nonetheless, C3a monocytes were enriched in functions, such as leukocyte migration and the endoplasmic reticulum stress response, in contrast to C3b monocytes which were associated with antigen processing, presentation, and detoxification, indicating that C3a is more active in cell transportation (Figure S12, Supporting Information). RNA velocity analysis predicted that C5 monocytes gave rise to two different cell lineages, with C8 monocytes serving as a major origin of Itgal^+^ macrophage (C7), and C3a committed to C11 monocytes and DIMs (C13 and 14). Conversely, MHCII^+^ macrophages and CAMs were not derived from monocytes, which was consistent with prior research indicating that they originate from embryonic yolk sac precursors, together with microglia.^[^ ^63^ ^]^ We also found that MHCII^+^ macrophages were enriched in genes associated with antigen processing and presentation functions, whereas CAMs were enriched in pathways related to macrophage or monocyte migration, revealing a crosstalk between CNS‐resident and peripheral macrophage subtypes (Figure S12, Supporting Information). In summary, we suggest that different macrophage subtypes have dichotomous tissue origins: Itgal^+^ macrophages and DIMs are monocyte‐derived, whereas CAM and MHCII^+^ macrophages may stem from embryonic precursors. To further discern the tissue origin of the DIMs, we extracted them alongside C3a, C3b, and C11 monocytes, and computed their velocity pseudo‐times. Our observation revealed that the CBM responded quickest to DIM differentiation among all four analyzed tissues (Figure 5D), which was further validated by cumulative probability distribution analysis (Figure 5E). Accordingly, we found that arginase 2 (*Arg2*) and guanine deaminase (*Gda*) played pivotal roles in modulating monocyte differentiation into DIMs (Figure 5F). Considering that DIMs most likely originate from C3a monocytes, we hypothesized that the C3a composition in the CBM would differentiate into cerebral DIMs post‐stroke. These results are consistent with previous reports that Itgal^+^ macrophages and DIMs originate from bone marrow monocytes but not from CAMs.^[^ ^66^ ^]^

We then proceeded to detect the potential sources of microglial subtypes. Although we could not include precursors of microglia owing to the limitation of mouse age, RNA velocity analysis revealed that the microglia had various immature states (C16 and C9) (Figure 5C). Notably, the C9 DAM, believed to be generated from mature microglia in neurodegenerative diseases, exhibited a transcriptional profile resembling immature microglia, in accordance with their “fetal like reprogramming” observed in Alzheimer's disease.^[^ ^66^ ^]^ Moreover, we identified a series of genes whose up‐ or down‐regulation might influence the maturation of various microglial subtypes (Figure 5G). Among them, *Wnk1* and *Tcerg1* were upregulated in DAM and might modulate the maturation of this stroke‐induced microglial subtype. The Cl^−^ transmembrane transporter modulates WNK1‐mediated SPAK/OSR1‐CCCs activation and promotes the survival and M2‐like polarization of microglia in ischemic stroke.^[^ ^72^ ^]^ Following stroke, microglial subtypes were enriched with genes associated with cell migration, chemotaxis, and biogenesis of organelles, in contrast to genes enriched with antigen processing or cell‐mediated immunity functions in sham mice, indicating an active transcriptomic and functional switching of microglia after stroke (Figure S12, Supporting Information).

Following stroke, shared and distinct transcription factors or pathways modulated the differentiation and function of DIMs and DAM (Figures S13 and S14, Supporting Information). Despite the presence of some shared transcription factors, distinct regulatory networks highlighted their specialized roles in post‐stroke immune responses and repair processes (Figure S13, Supporting Information). To investigate cell type specific functions, we investigated the spatiotemporal distribution of the three cell subpopulations (DIM, DAM and Neut_Cd14). Using the SPOTlight algorithm,^[^ ^73^ ^]^ we aligned our data with the spatial transcriptomic data provided by Zucha et al.^[^ ^32^ ^]^ and Han et al.^[^ ^33^ ^]^ The results revealed that DIMs represented the largest cell proportion 3 days post‐stroke and were distributed primarily in the penumbra, whereas DAM showed relatively high cell count on day 3 and 7 post‐stroke, implying its functions are in chronic tissue repair or gliosis (Figures S15 and S16, Supporting Information). In contrast, number of Neut_Cd14 peaked at 24 h in the infarction core, and diminished quickly afterward, indicating its role in acute‐phase immune responses. These findings highlight the temporal and spatial coordination of these immune cell subsets, each contributing to different stages of stroke‐induced inflammation and recovery.

Collectively, our analyses suggest that DIMs and DAM originate from the cranium and brain, respectively. We also provide insights into the regulatory genes and functions of specific monocytes/macrophages and microglial subsets.

## 3 Discussion

Although scRNA‐seq studies have expanded our knowledge of post‐stroke immune responses,^[^ ^13^, ^30^, ^74^ ^]^ the panorama and origin of brain‐resident and infiltrating immune cells during the acute phase of stroke remain elusive. In this study, we provide a cell‐resolution atlas that captures the transcriptomic characteristics of two bone marrow sites (CBM and FBM), PBMCs and brain tissue 24 h post‐stroke. In addition, we employed a p‐MCAO model, which was recommended by Stroke Therapy Academic Industry Roundtable (STAIR), to perform preclinical researches.^[^ ^75^ ^]^

While many studies emphasize the role of peripheral blood and peripheral bone marrow in post‐stroke inflammation,^[^ ^22^, ^76^ ^]^ emerging evidence suggests that the skull also contributes myeloid cells and B cells to the brain via the crania‐meninges‐brain route.^[^ ^18^, ^19^ ^]^ After comparing the cranium and peripheral bone marrow, we showed that neutrophils, followed by monocytes/macrophages, exhibited the greatest tissue variance after ischemic injury. Thus, we first focused on neutrophils’ annotation.

Among the six neutrophil subtypes, we identified a novel Neut_Cd14 subtype, characterized by high *Cd14* expression in cerebral neutrophils, indicating tissue‐specific tropism. We propose that CD14^+^ neutrophils originate from the CBM, based on transcriptome similarity and pseudotime analyses. Functional enrichment analyses indicate that Neut_Cd14 likely participates in phagocytosis and chemotaxis in the ischemic brain, consistent with neutrophils' known roles in protease release and extracellular trap formation.^[^ ^77^, ^78^ ^]^ Furthermore, we identified the key TF modules, including *Bhlhe40*, *Maff*, *Tgif1*, *Fosl1*, *Maf*, which, along with their predicted target genes, promote Neut_Cd14 differentiation. These TFs and their signaling pathways may serve as potential targets for clinical intervention or inflammation modulation.

Next, we studied monocytes/macrophages which showed the second‐highest tissue variance, and are another important branch of myeloid cell influx into the brain. Similar to neutrophils, various monocytes/macrophages subtypes have distinct location preferences. Among all monocytes/macrophages subsets, DIMs emerged predominantly in the brain (94.1%), especially after stroke. Previously, DIMs were reported to be linked to proinflammatory responses and immune activation in Alzheimer's disease (AD), or aging mouse models.^[^ ^66^ ^]^ However, their presence in the post‐stroke brain suggests they also play a role in acute neurological diseases. Compared to other monocytes/macrophages subtypes, DIMs showed elevated expression of *Il1b*, *Sell*, and *Cxcl3*, which encoded IL‐1β, Selectin L, and CXCR2 ligand, respectively, and were important for proinflammation,^[^ ^79^ ^]^ myeloid cell migration and chemotaxis,^[^ ^80^ ^]^ altogether underlying the proinflammatory roles DIMs might play. Our study also explores the ontogeny of different monocytes/macrophages subtypes. CAMs and monocyte‐derived macrophages display divergent differentiation pathways. Monocytes contribute CD11a^+^macrophages to peripheral blood and supply DIMs to the brain, whereas CAMs and MHCII^+^ macrophages may originate from embryonic cells. Moreover, we investigated the potential signals or genes that regulate DIM maturation and found that *Arg2* and *Gda* modulate this process. Mitochondrial *Arg2* regulates arginine metabolism and is essential for IL‐10 metabolic reprogramming of inflammatory macrophages.^[^ ^81^ ^]^ Glycodelin A (*Gda*) encodes a lipocalin that modulates monocyte deletion or polarization in peripheral organs, with its roles in bone marrow or brain still to be confirmed.

We also studied brain‐resident microglial cells, which shared overlapping transcriptomic features with CAMs. After vascular occlusion, microglia are mobilized in the penumbra immediately, even before neuron apoptosis occurs.^[^ ^82^ ^]^ One day after stroke, homeostatic microglia differentiate into either proinflammatory or anti‐inflammatory phenotypes. Consequently, microglia can play either detrimental roles in exacerbating ischemic injury or beneficial roles in neurogenesis, angiogenesis, and neuroprotection.^[^ ^83^, ^84^, ^85^, ^86^ ^]^ In this study, we successfully defined 8 microglial subclusters, and identified DAM subtype in stroked mice, which was recognized for its protective roles in AD.^[^ ^68^ ^]^ Intriguingly, DAM also presented immature transcriptional features in RNA velocity analysis, showing a similar gene expression pattern to the CD11c^+^ P7 youth‐associated microglial subtype, as reported by Silvin et al.^[^ ^66^ ^]^

However, this study had several limitations. First, we did not investigate all myeloid or lymphoid cell types, such as DCs or mast cells, due to their limited cell number, or B and T cells, which were involved at later stages of the post‐stroke immune response. In addition, while the potential functions and spatiotemporal distribution of newly defined myeloid cell subtypes have been studied, future experiments are warranted to fully elucidate their mechanisms and influence on stroke.

## 4 Conclusion

Collectively, we identified the transcriptomic features of brain‐resident immune cells and migrating myeloid cells during the acute phase of stroke. We successfully defined Neut_Cd14 and DIMs in the ischemic brain of mice and predicted that both cell types stemmed from the CBM, potentially contributing to the acute neuroinflammation and secondary brain injury post‐stroke. Moreover, we annotated DAM in the ischemic brain, which might originate from the homeostatic microglia.

## 5 Experimental Section

### Ethical Statement

All experiments with mice were performed in accordance with the Animal Care and Use Committees guidelines set by Capital Medical University. Protocols were approved by the Animal Care and Use Committee of Capital Medical University (approval ID: 202 001 006).

### Study Design

The first objective of this study was to explore the transcriptomic landscape of cerebral immune cells, especially myeloid cells and brain‐resident macrophages or microglia, and immune cells from their potential origins, namely the skull bone marrow, peripheral blood, and remote bone marrow (the femur was chosen to represent remote hematopoietic organs generating the most immune cells in peripheral blood) at the single‐cell level. Another main aim of the study was to detect the potential origin of acutely immigrated myeloid cells post‐stroke based on their gene expression signatures. For this purpose, a total 83 047 cells were sequenced from these four tissues and annotated their corresponding cell types. Male mice aged 10 to 12 weeks were sacrificed. The gene expression features of different immune cell types between the CBM and FBM were then compared, with a focus on the top two cell types that exhibited the greatest tissue variance. A new neutrophil subtype (Neut_Cd14) was identified in the brain after stroke. Flow cytometry (*n* = 5) and qPCR (*n* = 4) were utilized to prove its existence and “location preference”. Furthermore, the cerebral neutrophil origin was investigated based on transcriptomic similarities among neutrophil subsets from four tissues. The dataset was integrated with other neutrophil datasets (Bouyer, Xie, and Kolabas) to investigate the accuracy of the subtype annotation strategy and tissue origin analyses. To identify the underlying regulons that modulate the differentiation of neutrophil subtypes, pySCENIC was used to extract key transcription factors and discern their target genes. Heterogeneity and homogeneity among different subtypes of microglia and monocytes/macrophages were also explored. Differentially expressed genes were identified by comparing the different experimental conditions and tissues. The RNA velocities of the different macrophage subtypes were calculated to infer their cell states and potential origins. The spatiotemporal distribution, potential functions and modulators of stroke‐responsive myeloid and brain‐resident immune cells were also studied.

### Statistical Analysis

Three independent mice were pooled in both p‐MCAO and sham groups for single cell isolation and scRNA‐sequencing. Pre‐processing of the raw sequencing data involved alignment to the mouse reference genome (mm10) and estimation of cells and UMIs using Cellranger. Quality control included filtering cells with gene counts < 200 or > 6000 and mitochondrial reads > 20%, followed by duplicate removal using Scrublet. Downstream analysis was performed using Seurat (V4.1.0). Seurat's NormalizeData and FindVariableFeatures functions were used to normalize the data and identified 2000 highly variable genes to address sequencing depth bias. Multiple single‐cell sequencing samples were integrated using CCA to mitigate batch effects, and correlation analysis was performed using Pearson's correlation. Cell type and subcluster identification were performed using the ScaleData function, followed by PCA and UMAP for dimensionality reduction and clustering using FindCluster. Differential analysis relied on log2FC and two‐sided Wilcoxon rank sum tests with the Bonferroni correction for comparisons between groups. Flow cytometry data were collected using a Cytoflex S flow cytometer and analyzed using FlowJo software. Data that adhered to a normal distribution were presented as mean ± standard error of mean (SEM). Paired t test was utilized to compare data from p‐MCAO and sham groups. One‐way analysis of variance (ANOVA) followed by Tukey's post‐hoc test was used to compare data from three or more groups with one variable. A confidence interval of 95% was applied, and statistical significance was set at *p* < 0.05. Data were analyzed using R (version 4.2) and GraphPad prism 9.5. Details of the statistical tools, methods, and thresholds are provided in the Supporting Information.

### Ethics Approval Statement

All protocols used in this study were approved by the Animal Care and Use Committees of Capital Medical University.

## Conflict of Interest

The authors declare no conflict of interest.

## Author Contributions

M.Y., Y.L., and K.S. contributed equally to this work. Y.W. and M.L. conceptualized the study. The methodology was developed by K.S., M.Y., Y.L., Y.W., and M.L. Visualization was carried out by Y.L., S.M., M.L., and X.W. Funding acquisition was handled by Y.W., M.L., and K.S. Project administration was managed by M.Y., Y.L., K.S., Y.W., M.L., and X.W. Supervision was provided by Y.W., M.L., and X.L. The original draft of the manuscript was written by M.Y., Y.L., K.S., X.H., X.L., and X.W. S.M., X.L., M.L., Y.W., and F.D.S. were responsible for the review and editing of the manuscript.

## Supporting information



Supporting Information

## Data Availability

The data that support the findings of this study are available on request from the corresponding author. The raw sequence data reported in this paper have been deposited in the Genome Sequence Archive (GSA: CRA016580) that are publicly accessible at https://ngdc.cncb.ac.cn/gsa.
