# Supplementary material for: Single‐Cell Transcriptomes of Immune Cells from Multiple Compartments Redefine the Ontology of Myeloid Subtypes Post‐Stroke
Source: Adv Sci (Weinh). 2025 Feb 11;12(13):2408722. doi: 10.1002/advs.202408722 (PMC11967789; doi:10.1002/advs.202408722)
Supplement: Supplementary file 1 — Supporting Information [file ADVS-12-2408722-s001.pdf]

ADVANCED SCIENCE Open Access

Supporting Information

for *Adv. Sci.*, DOI 10.1002/advs.202408722

Single-Cell Transcriptomes of Immune Cells from Multiple Compartments Redefine the Ontology of Myeloid Subtypes Post-Stroke

Mo Yang, Yixiang Li, Kaibin Shi, Xuezhu Wang, Xiangrong Liu, Xiang Huang, Fu-Dong Shi, Shaojie Ma, Mingfeng Li and Yilong Wang**

Supplementary Materials for

Single-Cell Transcriptomes of Immune Cells from Multiple Compartments Redefine the Ontology of Myeloid Subtypes Post-Stroke

The PDF file includes:

Materials and Methods

Fig. S1 to Fig. S16

Table S1 to Table S3

References [36,44,73,87-93]

MATERIALS AND METHODS

Rodents and pMCAO model induction

C57BL/6J mice were purchased from Beijing Vitalriver Co., Ltd and housed at least one week with local colonies prior to experiment. Adult male mice aged 10-12 weeks were used in this study. All mice were bred and housed under standardized 12-hour light/dark cycle in controlled environment with consistent temperature (23±1°C) and humidity (50–60%), with no more than five animals each cage. All protocols used in this study were approved by the Animal Care and Use Committees of Capital Medical University.

Above adult mice (weighted 22-26 g) were randomly assigned to either p-MCAO or sham groups. Animals were anesthetized by intraperitoneally injection of chloral hydrate (30 mg/kg). Mice body temperature was maintained at 37.0 ± 0.5 °C with heating pad throughout the operation. In brief, the mice were fixed at supine position, and 38midline incision was made. Next, we separated and ligated right external carotid artery and common carotid artery separately. The right MCA was further occluded by inserting a heat-rounded 6/0 nylon suture from a superior cut above the common carotid artery ligation site. Longa neurological function evaluation and Laser Doppler flowmetry were applied to assure successful MCA occlusion. Sham group mice were subjected to same operation procedure without nylon suture insertion.

Tissue preparation and cell isolation

24 hours post pMCAO induction, blood from retroorbital sinus was collected rapidly after anesthesia for p-MCAO and sham mice. Animals were then perfused with ice-cold PBS. PBMC was isolated from blood samples using Ficoll-Paque separation media. Skull and brain were harvested immediately after decapitation posterior to the occipital bone. The dura mater was further peeled from the inner skull utilizing fine tissue forceps under a microscope. Skull samples were mechanically chopped up and incubated in FACS buffer (cold PBS with 1% BSA). After several gentle pipettes, we filtered cranial bone marrow cells through a 70-um cell strainer. Besides, we also collected bilateral femur from the same mouses. Muscle and tendon tissue was removed from femur, and the bone marrow cells were flushed and filtered also through a 70-um cell strainer. Meanwhile, brain stem and cerebellum were removed from brain tissues. Brain was minced and

digested in pre-warmed digestion buffer (EBSS containing 1 vial/5ml papain (Worthington), collagenase I (Sigma-Aldrich), DNase I (Sigma-Aldrich), 1% BSA) at 37°C for 30 minutes. During incubation, brain was gently triturated with a wide-bore 1ml pipette every 10 minutes. Enzymes were later inactivated by adding an equal volume of PBS. The mixture was then centrifuged at 2000rpm for 5 min at 4°C. Cell pellets were resuspended in 30% Percoll solution. Next, we centrifuged the solution at 400g for 30 minutes to remove the resulted upper myelin-containing layer. After another round of PBS wash, we added in CD45 antibodies (Biolegend) in PBS resuspended solution and incubated the mixture for 30 minutes. To achieve better cell viability, we utilized CD45 MicroBeads from Miltenyi Biotec other than flow cytometry to select brain immune cells.

Flow cytometry

Single cell suspensions from peripheral blood, brain, femor and skull were prepared as above and resuspended in FACS buffer. Fluorescence-conjugated anti-mouse antibodies were then added to label neutrophils or specific subtype of neutrophils: CD45, CD11b, Ly-6G and CD14 (all from Biolegend). Cells and antibodies were incubated for 30 minutes on ice. After staining, cells from 4 tissues were washed twice in PBS and resuspended in FACS buffer. Cells were acquired using Cytek NL-CLC3000 (Cytek Biosciences) flow cytometer. Finally, data were analyzed using FlowJo_V10.

RNA purification, cDNA synthesis, and qPCR

We isolated Ly6g+ murine neutrophils from brain, femur, skull and peripheral blood tissues using MicroBeads from Miltenyi Biotec. RNA extraction and reverse transcription of neutrophils were performed via a UniCell to cDNA Synthesis SuperMix following manufacturer's recommendations. The cDNA was subjected to qPCR on a ViiA 7 Real-time PCR System (Applied Biosystems). GAPDH was used as a normalizer to calculate ΔCt. The primers' sequences are listed in Table S3.

cDNA library construction and single-cell RNA sequencing

First, we counted cell number by Countess™ II Automated Cell Counter as well as

hemocytometers with trypan blue. The cell viability was above 90%. Brain, femur, skull and PBMC cells from three independent mice were pooled together to reduce sample-to-sample variation. Abundant or limited single cell suspensions from above 4 tissues were concentrated or diluted to achieve a cell concentration of 10,000 cells/ml. The cell suspension was loaded into Chromium microfluidic chips with 30 v3 chemistry and further barcoded with a 10× Chromium Controller (10X Genomics). RNA from above cells was reverse-transcribed and cDNA libraries constructed with reagents from a Chromium Single Cell 30 v3 reagent kit (10X Genomics) according to the manufacturer's instructions. We subsequently performed single-cell sequencing with Illumina NovaSeq 6000 according to the Illumina's instructions.

Statistical analysis

(1) Pre-processing of raw sequencing files

The raw data was aligned to the mouse reference genome (mm10) and analyzed using Cellranger to estimate cells and associated unique molecular identifiers (UMI). Duplicates were removed and the number of UMI tags for each gene was obtained. Downstream analysis was performed using Seurat (V4.1.0).[87] To remove low-quality cells and soluble RNA, we filtered out cells with gene expression counts < 200 and > 6000. At the same time, we also removed cell samples containing 20% mitochondrial sequence. After removing double cells by Scrublet,[88] high-quality cells were finally obtained. The NormalizeData function was used to remove the influence of sequencing depth, and the FindVariableFeatures function was used to find 2000 highly variable genes in each sample. Since this project involved multiple single-cell sequencing samples, sample data integration was required. The cells were integrated with the Canonical correlation analysis (CCA) algorithm to remove potential batch effects.

(2) Cell type and subcluster definition

The combined data were normalized using the ScaleData function. Principal component analysis (PCA) was performed on the normalized matrix using the RunPCA function. The Elbow plot is used to determine the appropriate dimension of the principal components, which are selected for UMAP dimension reduction to calculate the distance between cells. The cells were clustered using

the FindCluster function based on the nearest neighbor graph clustering algorithm. The cell type corresponding to each cell cluster was identified by calculating the genes that were significantly up-regulated in each cell cluster compared with other clusters, consulting the literature and VlnPlot and FeaturePlot functions. We defined a cluster to be an independent cell type or subtype only when cluster cell number equaled or exceeded 10. The correlation between the reference data and the cells to be identified was integrated, and the cells to be identified were named as the cell type with the highest correlation. The proportion of identified cell subsets was calculated to obtain cell number information. Pie charts and radar charts were drawn using ggplot2 (V 3.4.3) and ggrader (V 0.2), respectively.

(3) Identification of differentially expressed genes between groups

The identification of differentially expressed genes was performed using the FindMarkers function. Different significance thresholds were set for different difference comparisons (see the legend in the main text for details), but all differentially expressed genes met avg_log2FC > 0.1 and p_val_adj < 0.01. Volcano and Venn plots were created using ggplot2 (V 3.4.3) and Venn (V 1.11).

(4) Enrichment analysis of gene pathways

Pathway enrichment analysis was performed using the MSigDB function in the R package msigdbr (V7.5.1) with the following parameters: species = "Mus musculus", category = "C5", subcategory = 'BP'.

(5) Developmental trajectory inference

Pseudotime analysis was performed on neutrophils using Monocle2 (V 2.26.0). To identify differentially expressed genes among neutrophil subtypes, the FindAllMarkers function was used, and only genes with p_val_adj < 0.01 were considered. The top 200 genes were selected based on the value of avg_log2FC. Next, the DDRTree algorithm was used to reduce the data space to two dimensions. Then, the construction of the differentiation trajectory was completed using the reduceDimension function (with max_components = 2 and method = 'DDRTree') and the orderCells function.

(6) pySCENIC analysis

The pySCENIC package (V 0.12.1) was utilized to infer gene regulatory networks, investigate potential regulators for each neutrophil subtype, and calculate their corresponding Regulon activity in each cell.[44] The reference lists of transcription factors were compared to the genes in the single-cell expression matrix to identify co-expressed genes. Then, the cisTarget database (mm9-tss-centered-10kb-10species) was used to analyze the cis-regulatory motifs of the co-expression modules to obtain enriched motifs. The modules enriched by TF motifs and their corresponding target genes were kept. Based on the ranking of each motif in the motif-gene annotation file, the motif enriched by each module was calculated, the false positive or non-direct target genes were removed, and a total of 113 real TFs were identified.[89] All target genes regulated by the same transcription factor are combined into a Regulon, which is then merged with the single-cell expression matrix. The AUCell algorithm is used to score the regulatory activity of identified regulators in each cell. To calculate the Regulon specificity score (RSS) of a regulator in each neutrophil subtype, we used the calcRSS function. The RSS value reflects the specificity of the transcriptional activity of the regulator in a certain cell type. We used the R package ComplexHeatmap (V 2.12.0) to extract the top 10 characteristic genes, sorted by avg_log2FC, of the newly defined neutrophil subtypes and to draw heat maps for display. The differentially expressed genes in each subgroup, identified by TOP200, were crossed with transcription factors and target genes, and data of TF and targets importance >2 and Degree >2 were screened and brought into Cytoscape (V 3.10.1) for mapping.

(7) Scoring of biological processes

The AddModuleScore function of the Seurat package was used to score the gene sets regulating proliferation, metabolism, degranulation, apoptosis, chemotaxis, senescence, maturation, phagocytosis, and oxidative stress of the neutrophil sub-clusters, and the gene set characteristic score corresponding to each neutrophil subtype was obtained, to reflect the differences in biological functions of different neutrophil subtypes.[36,90] Then the Heatmap function and ggboxplot function are used to complete the heat map and boxplot.

(8) RNA velocity analysis

RNA velocity and latent time were estimated using ScVelo (V 0.3.0).[91] The fastq files for each experiment were processed into loom files for each sample using Velocyto (V 0.17.17). We then generate spliced and unspliced matrices for each sample in the Seurat file. RNA velocity was calculated using the relative ratio of unspliced and spliced mRNA abundances as an indicator of cell state. The velocity field is projected onto the UMAP graph. scvelo is then used to perform downstream analysis to identify highly dynamic genes, and the consistency measure between adjacent cells is calculated based on speed for pseudotime inference. We use pseudo-temporal traces to identify the predicted ancestors of individual cells and localize the directionality of partition-based graph abstractions (PAGA).

(9) CytoTRACE2 analysis

The CytoTRACE2 package (v1.0.0) is a method for predicting cellular potency and developmental potential from single-cell RNA-seq data.[92] The single-cell RNA-seq data object is passed to the cytotrace2 function for analysis, using raw count data and specifying the species as mouse. An annotation data frame is then created.

(10) Palantir analysis

Palantir is an algorithm for aligning cells along differentiation trajectories, capable of capturing the continuity and stochasticity in the cell differentiation process.[93] Convert the data into an AnnData object and perform analysis according to the Palantir standard process, calculate pseudotime, divide the pseudotime into 20 bins, calculate the proportion of different tissue subgroups within each pseudotime group, and plot the results.

(11) SPOTlight analysis

This experiment analyzes spatial transcriptomics data using the SPOTlight pipeline.[73] scRNA-seq data is preprocessed, selecting 3,000 highly variable genes and identifying marker genes for cell types, filtering those with an AUC value greater than 0.6. Next, a random sample of up to 100 cells is selected from each cell type. SPOTlight is then used for deconvolution analysis, matching single-cell data with spatial data, and employing the NMF model for training and deconvolution to extract the spatial distribution of cell types. The values corresponding to cell subpopulations

with proportions less than 0.1 in the matrix are replaced with zero, and the results are subsequently visualized.

Supplementary Materials

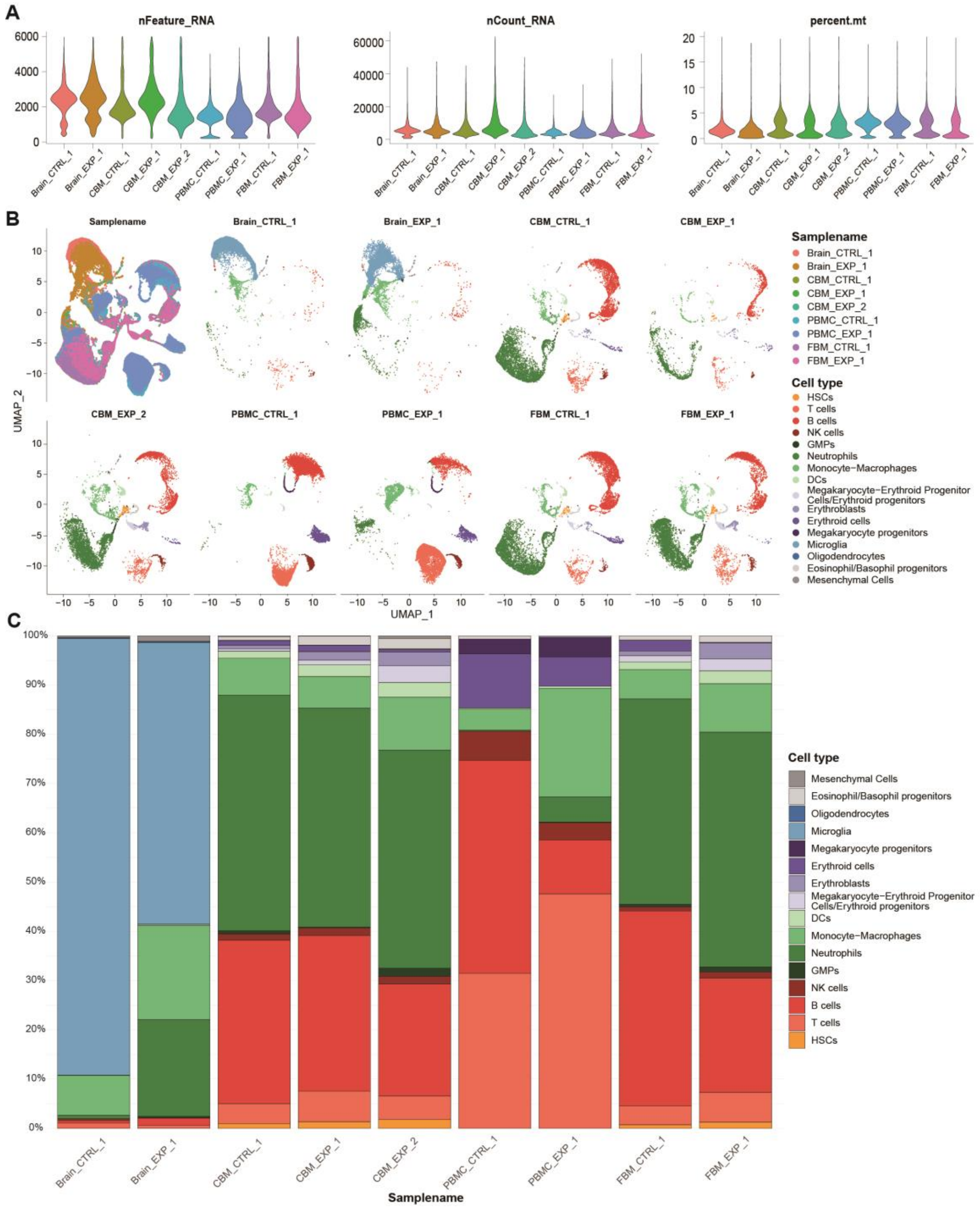

Fig. S1. Quality overview of this article's scRNA-seq data

(A) Violin plots showing the sample quality metrics generated in this article. Number of UMI per cell (nCount_RNA) (left) Number of genes detected per cell (nFeature_RNA) (middle) Ratio of mitochondria (percent. mt) (right).
(B) The UMAP diagram shows the source of the data. The UMAP information of each sample is displayed separately according to sample names.
(C) The bar chart shows the proportion of cell subsets in each sample.

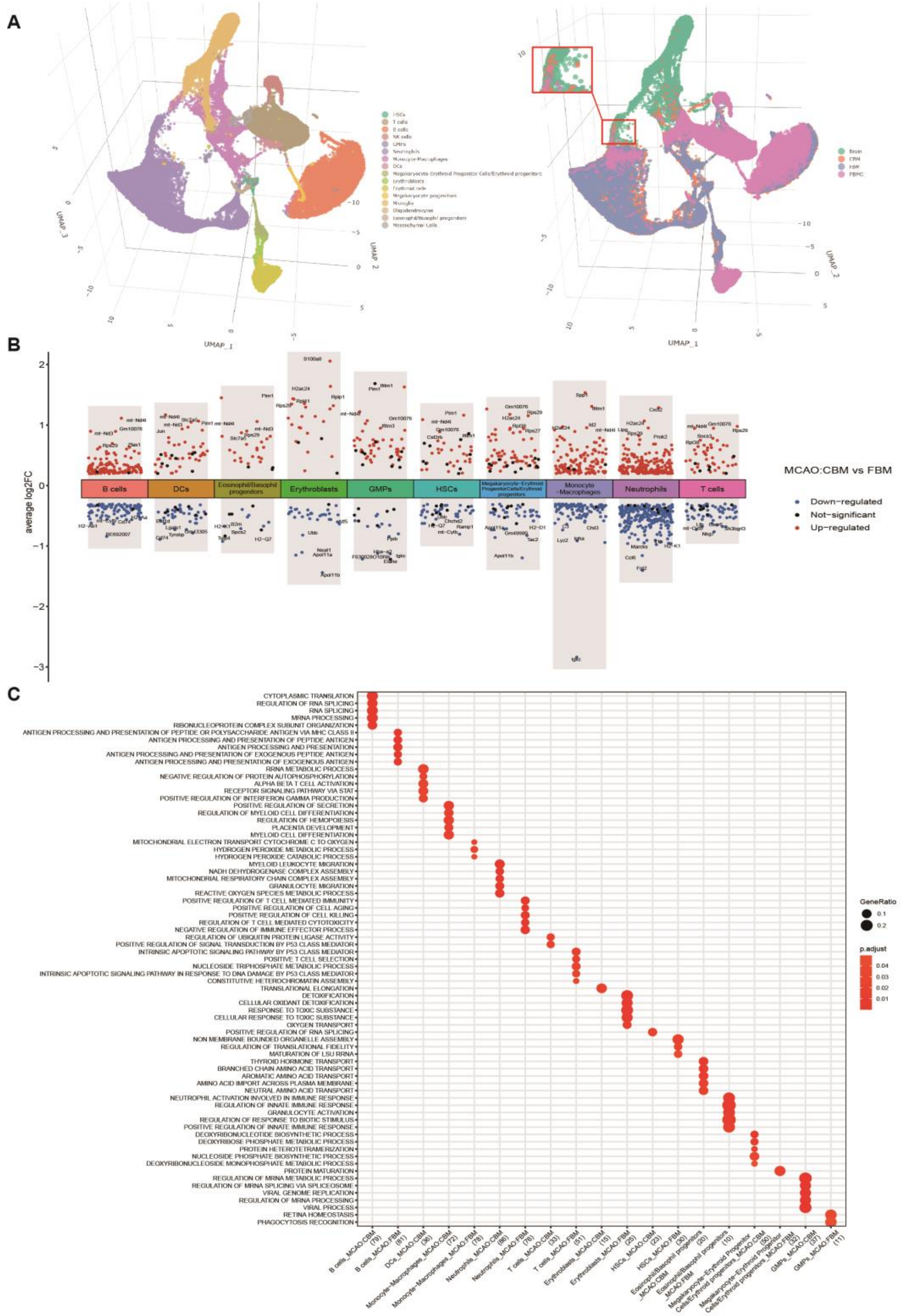

Fig. S2. Spatial relationships and gene expression analysis in CBM and FBM.

(A) The spatial relationships of each cell are presented using cell clusters (left) and tissue sources (right).

(B) Cell type-specific genes differentially expressed in CBM and FBM. Those with p_val_adj < 0.05 and abs(avg_log2FC) > 0.25 were filtered.

(C) GO functional analysis of the top 100 differentially expressed genes in each sub-cluster in B.

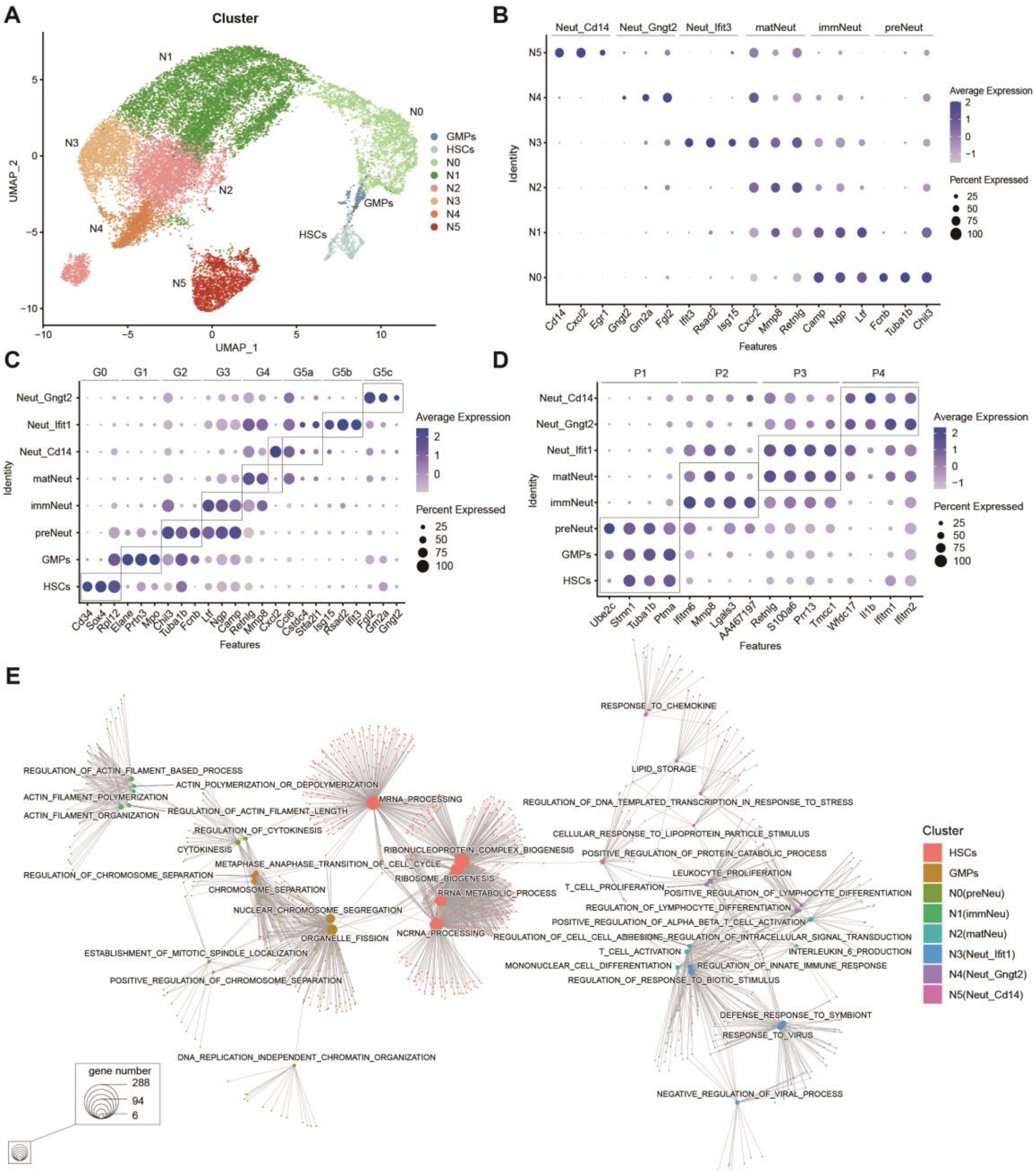

Fig. S3. Neutrophil subcluster analysis

(A) UMAP plot of six sub-clusters (N0-N5) resulting from the extracted neutrophil transcriptome.

(B) Dot plot of differentially expressed genes in the neutrophil subcluster selected for this dataset.

(C) Dot Plot of Differentially Expressed Genes Characterizing Neutrophil Subclusters as Defined by Xie et al.

(D) Dot Plot of Differentially Expressed Genes Characterizing Neutrophil Subclusters as Defined by Bouyer et al.

(E) GO function analysis in the neutrophil subcluster.

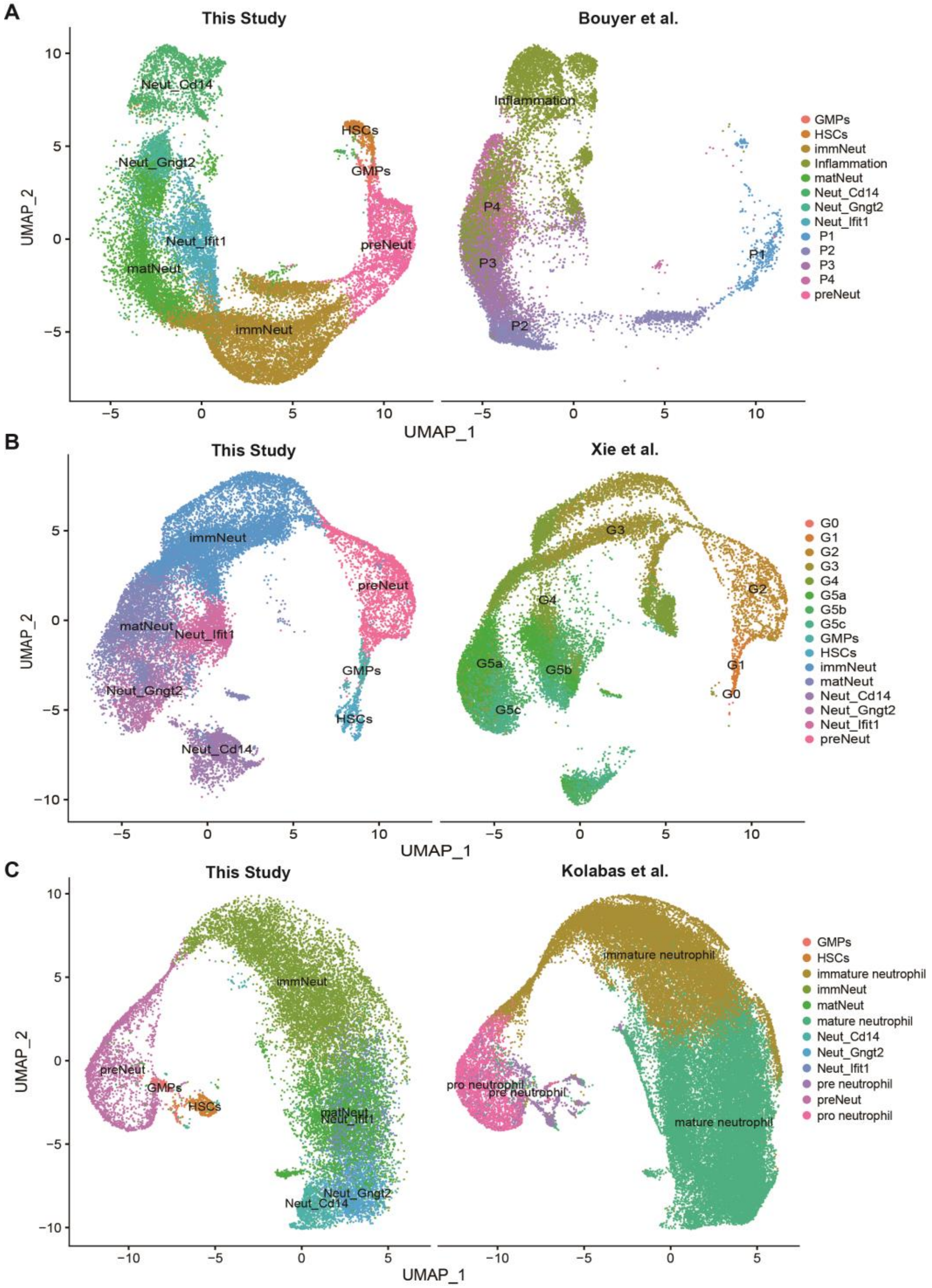

Fig. S4. Data integration and correlation comparison of neutrophil subsets.

(A) The CCA algorithm was used to integrate the neutrophil lineage data of this article and the neutrophil data of Bouyer et al. to draw UMAP and split it by sample name.

(B) The CCA algorithm was used to integrate the neutrophil lineage data of this article and the neutrophil data of Xie et al. to draw UMAP and split it by sample name.

(C) The CCA algorithm was used to integrate the neutrophil lineage data of this article and the neutrophil data of Kolabas et al. to draw UMAP and split it by sample name (left).

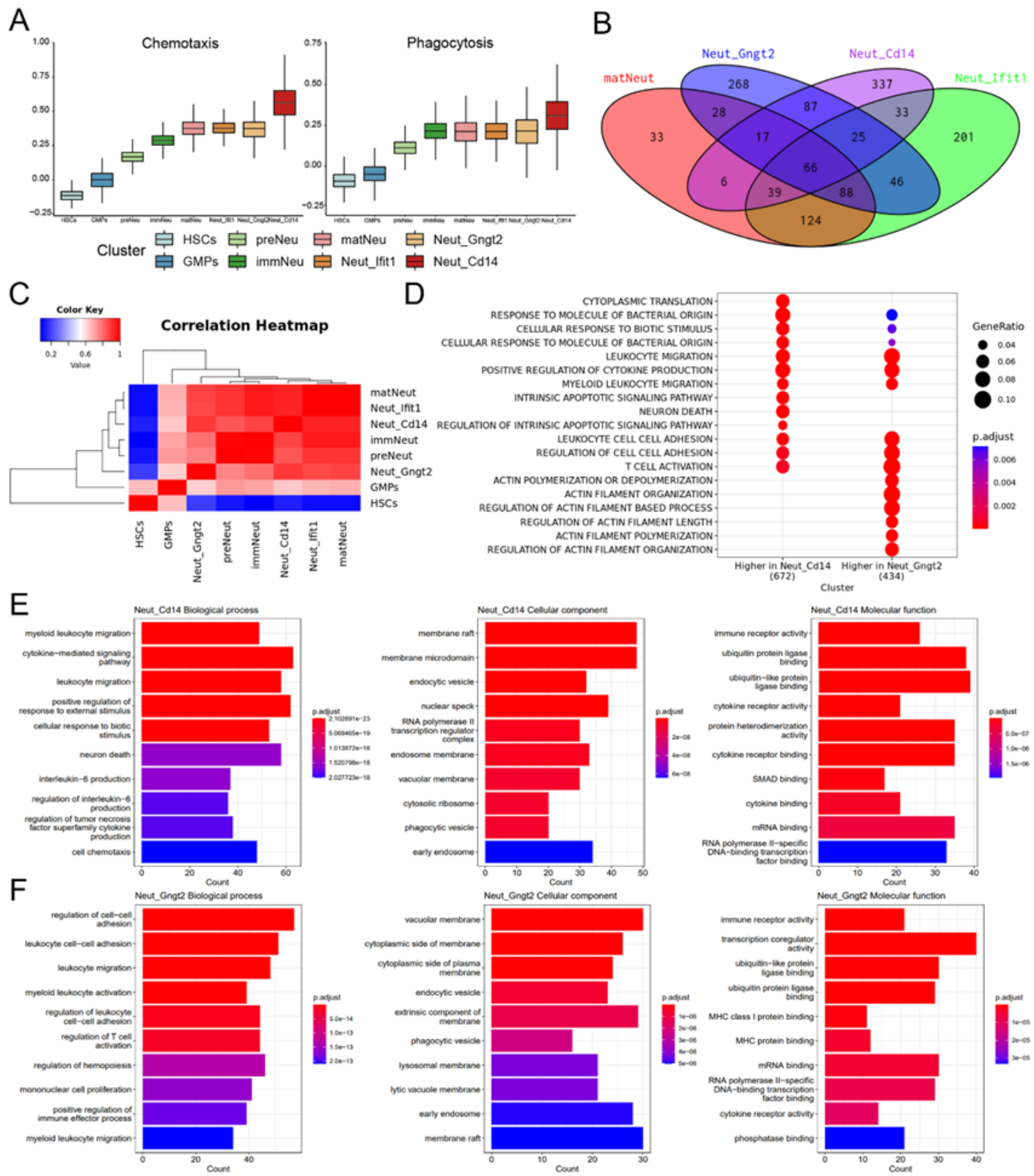

Fig. S5. Differential analysis of Neut_Cd14 and Neut_Gngt2.

(A) Box plot of functional scores of mature neutrophils in different regions from our data.

(B) Venn plot of differential gene expression between Neut_Cd14, Neut_Gngt2, Neut_Ifit1, and matNeut. (p_val_adj<0.05&avg_log2FC>0.25).

(C) Correlation analysis between subtypes.

(D) GO functional analysis of the specific regulatory factors of between Neut_Cd14 and Neut_Gngt2.

(E) GO functional enrichment analysis related to Neut_Cd14.

(F) GO functional enrichment analysis related to Neut_Gngt2.

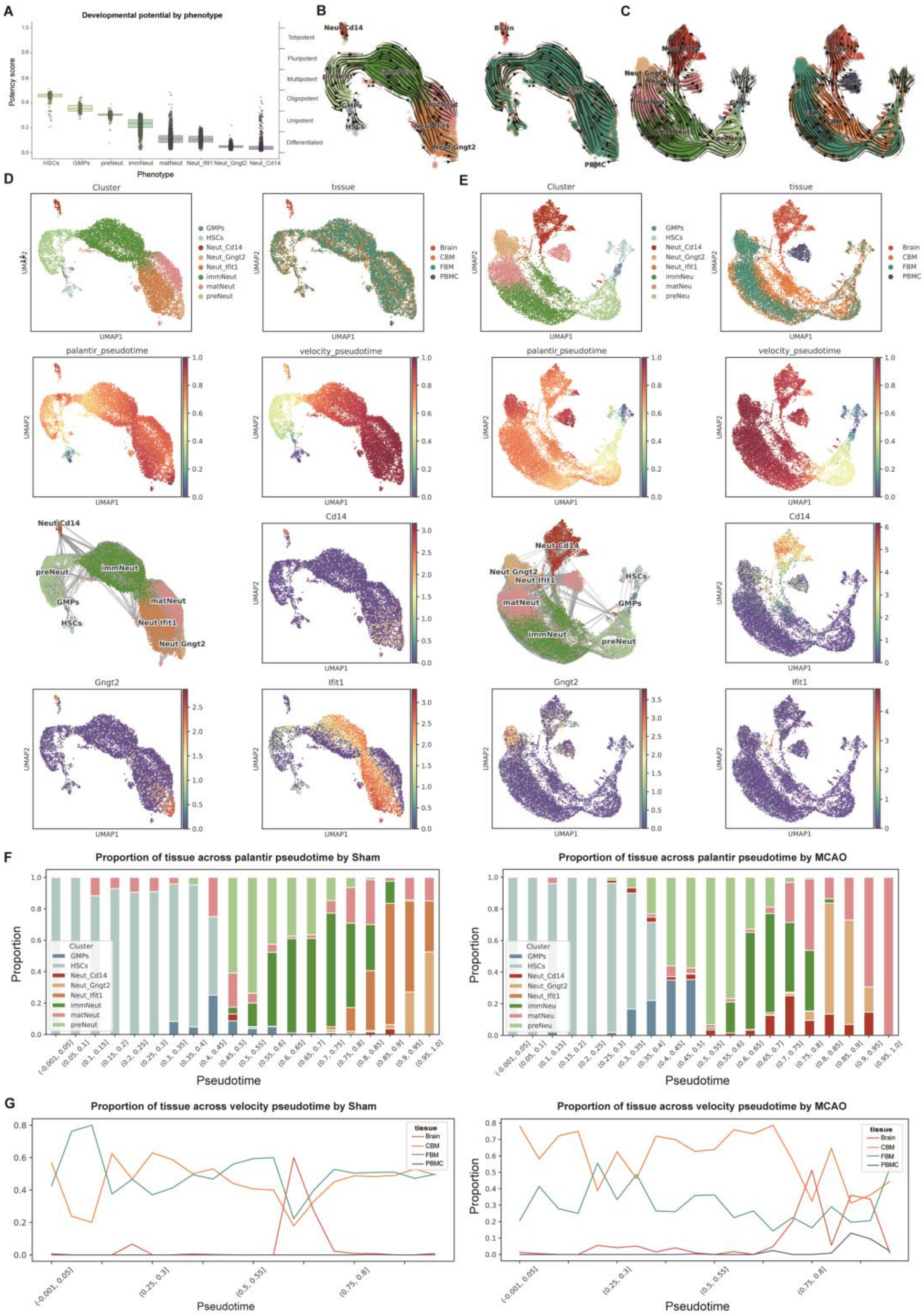

Fig. S6. Neutrophil pseudotime analysis

(A) Neutrophil differentiation maturity analysis using Cytotrace2.

(B) RNA velocity stream plots at Sham.

(C) RNA velocity stream plots at MCAO.

(D) Palantir and scVelo analysis of neutrophil differentiation in the Sham with differential gene expression on UMAP.
(E) Palantir and scVelo analysis of neutrophil differentiation in the MCAO with differential gene expression on UMAP.
(F) Proportion of tissue across palantir pseudotime by state. Division of pseudotime into 20 intervals, color-coded by celltype.
(G) Proportion of tissue across velocity pseudotime by state. Division of pseudotime into 20 intervals, color-coded by tissue.

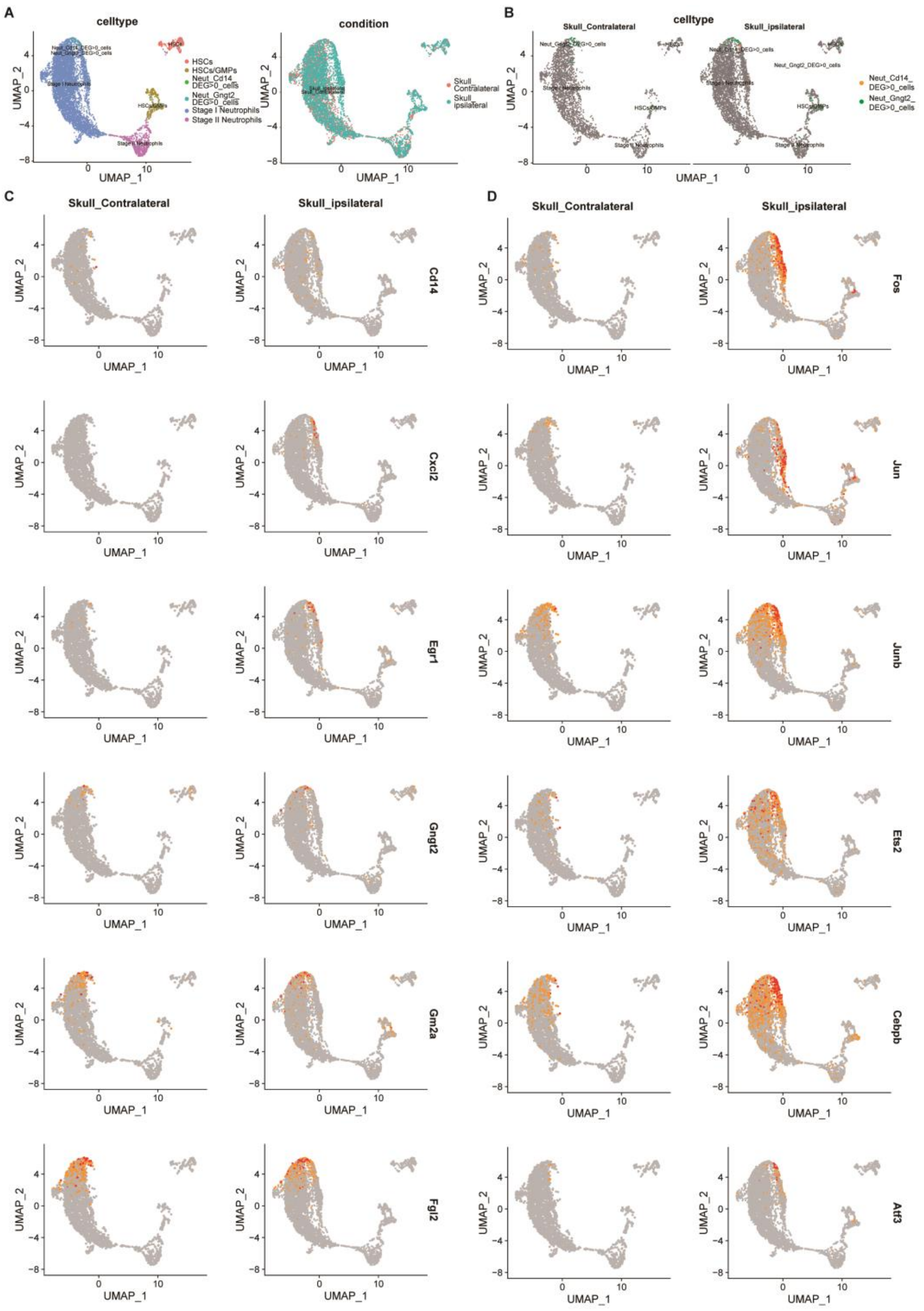

Fig. S7. Early immune response in the skull.

(A) UMAP plot of neutrophil data from Xu et al. based on celltype and state.

(B) UMAP plot of neutrophil data from Xu et al, categorized by tissue and color-coded by new cell type.

(C) UMAP plot of gene expression for Neut_Cd14 and Neut_Gngt2 markers.

(D) UMAP plot of Neut_Cd14 specific transcription factor expression.

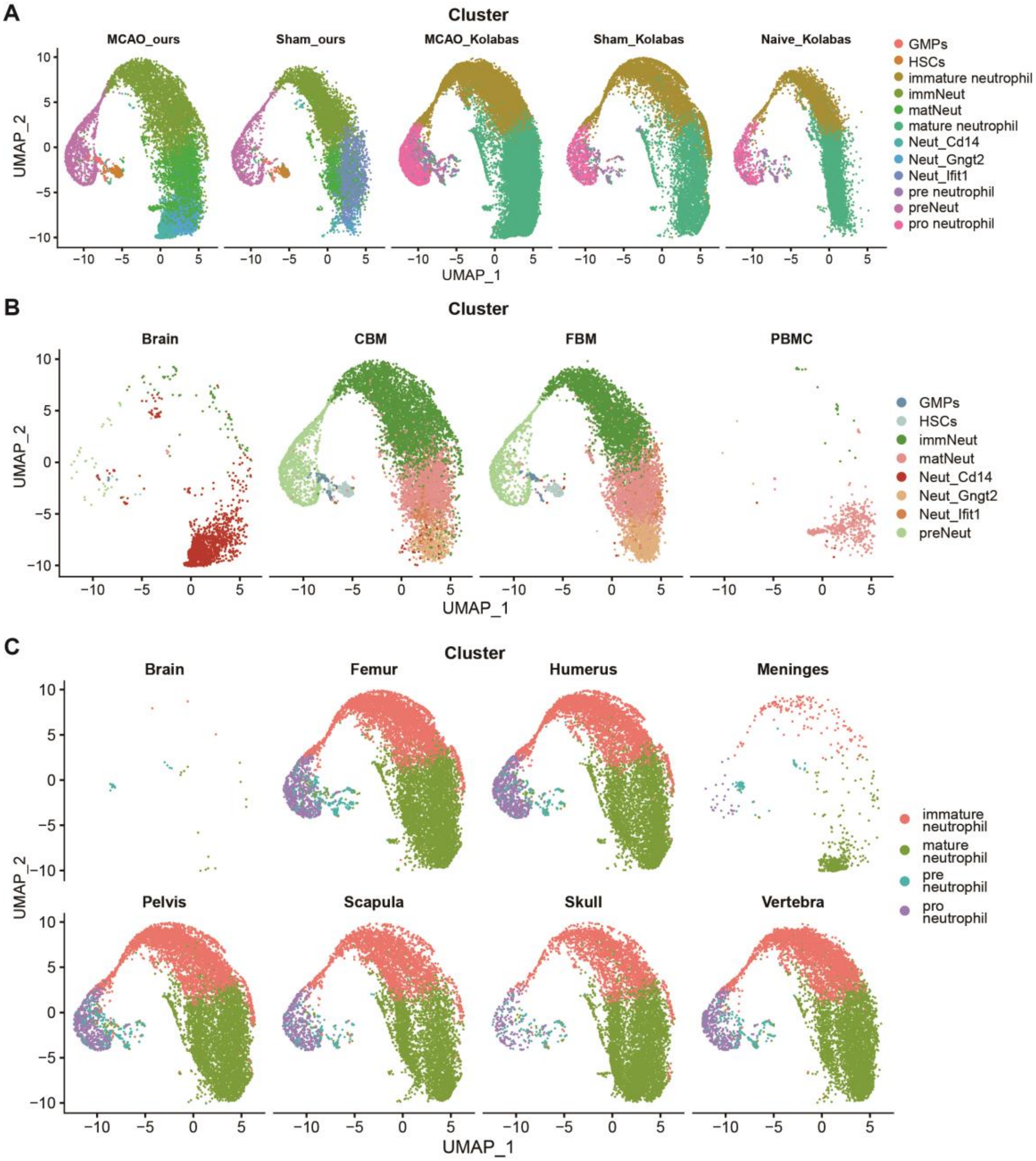

Fig. S8. Integrated UMAP showing neutrophil subclusters partitioned by experimental state and tissue

(A) The UMAP integrated with the neutrophil data of Kolabas et al. was displayed according to the experimental state partition.

(B) The integrated UMAP was partitioned by tissue to show the neutrophil subclusters defined by the data in this article.

(C) The integrated UMAP was partitioned by tissue to show the neutrophil subclusters as defined by the data of Kolabas et al.

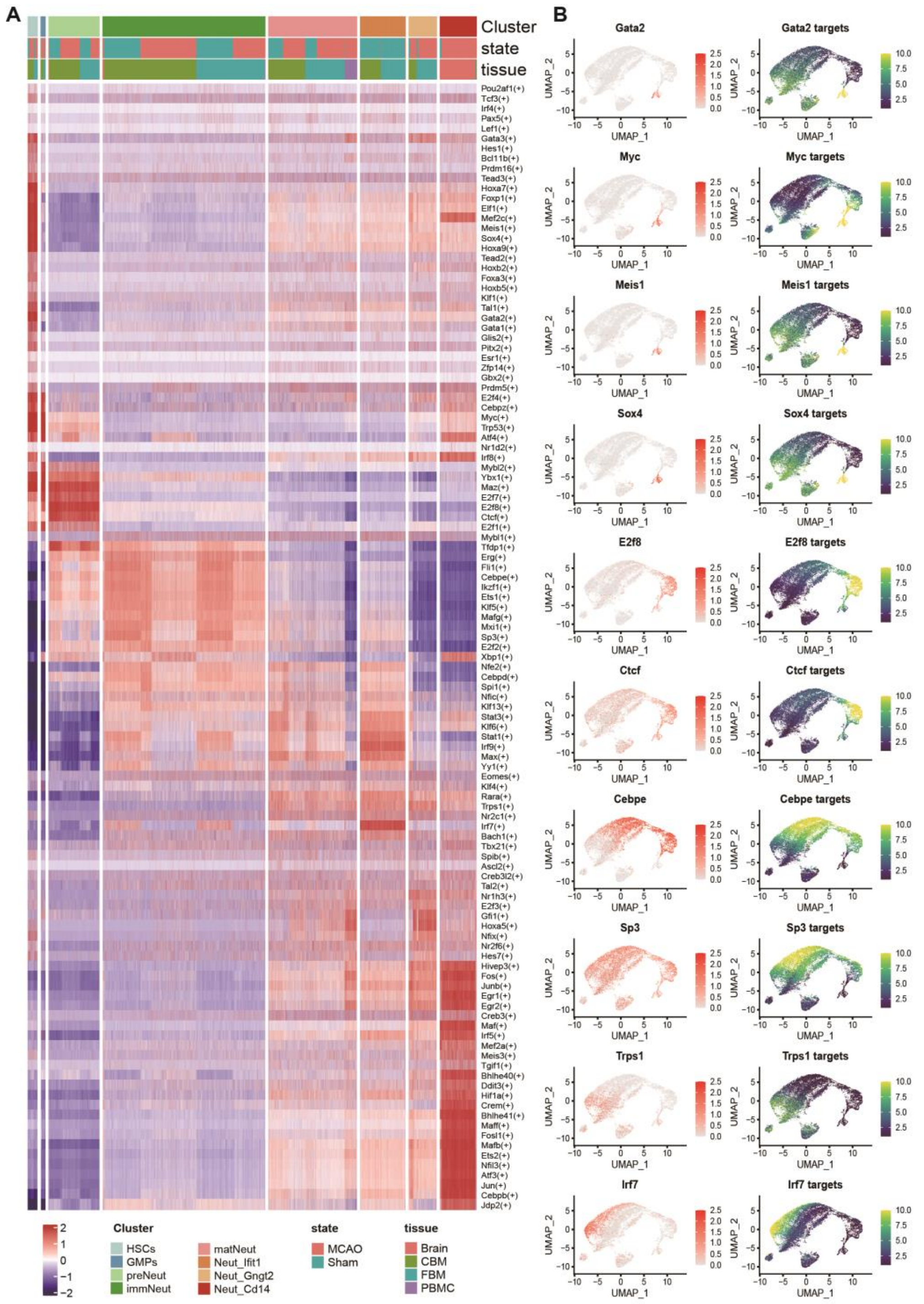

Fig. S9. Neutrophil transcriptional regulatory network and cellular function.

(A) ComplexHeatmap plot of 113 regulatory factors.

(B) UMAP plot showing the distribution of highly active TFs and their target genes in the other subcluster.

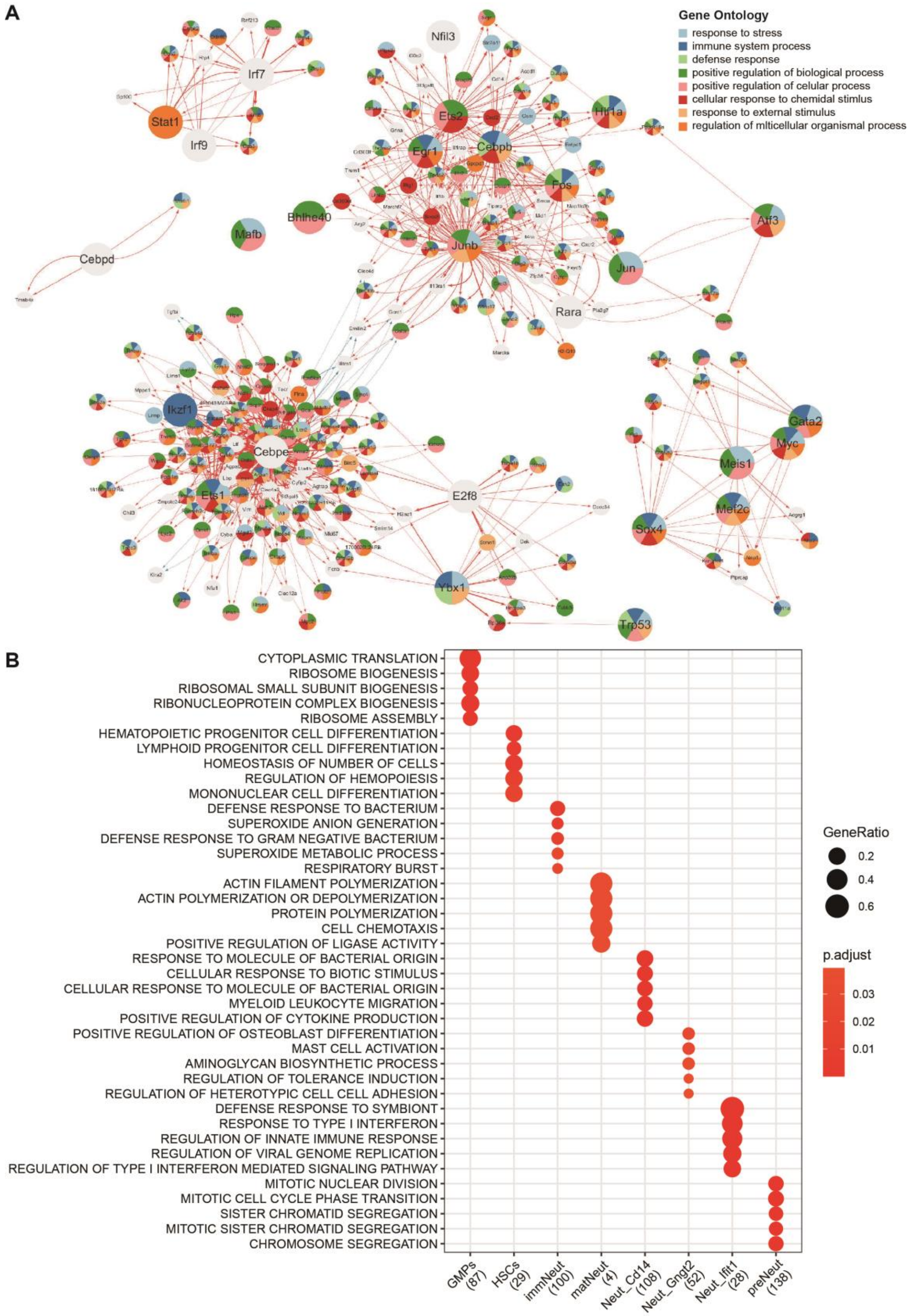

Fig. S10. Specific functional analysis of transcription factors and target genes in each subcluster.

(A) Predicted TF regulatory network with nodes colored by Gene Ontology.
(B) GO functional analysis of the specific regulatory factors of each neutrophil subcluster.

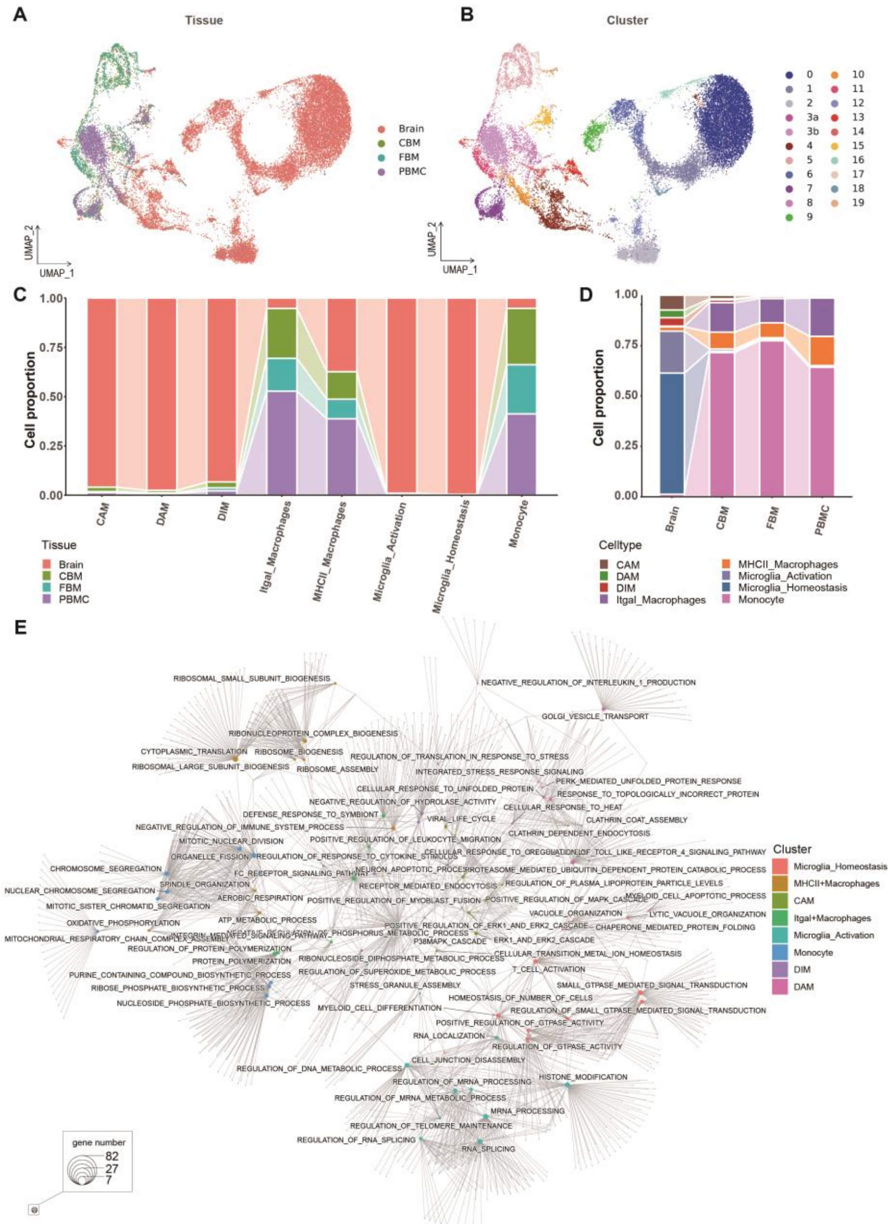

Fig. S11. Reclassification of monocyte-macrophages and microglia
(A) UMAP map of monocyte-macrophage and microglial aggregates colored by tissue expression.
(B) UMAP map of monocyte-macrophage and microglial aggregates colored by tissue expression.
(C) The proportion of cells of different tissue in each cell subcluster.
(D) The proportion of cells in different cell subclusters in each tissue.

(E) GO function analysis in the monocyte-macrophages and microglia subcluster selected for this dataset.

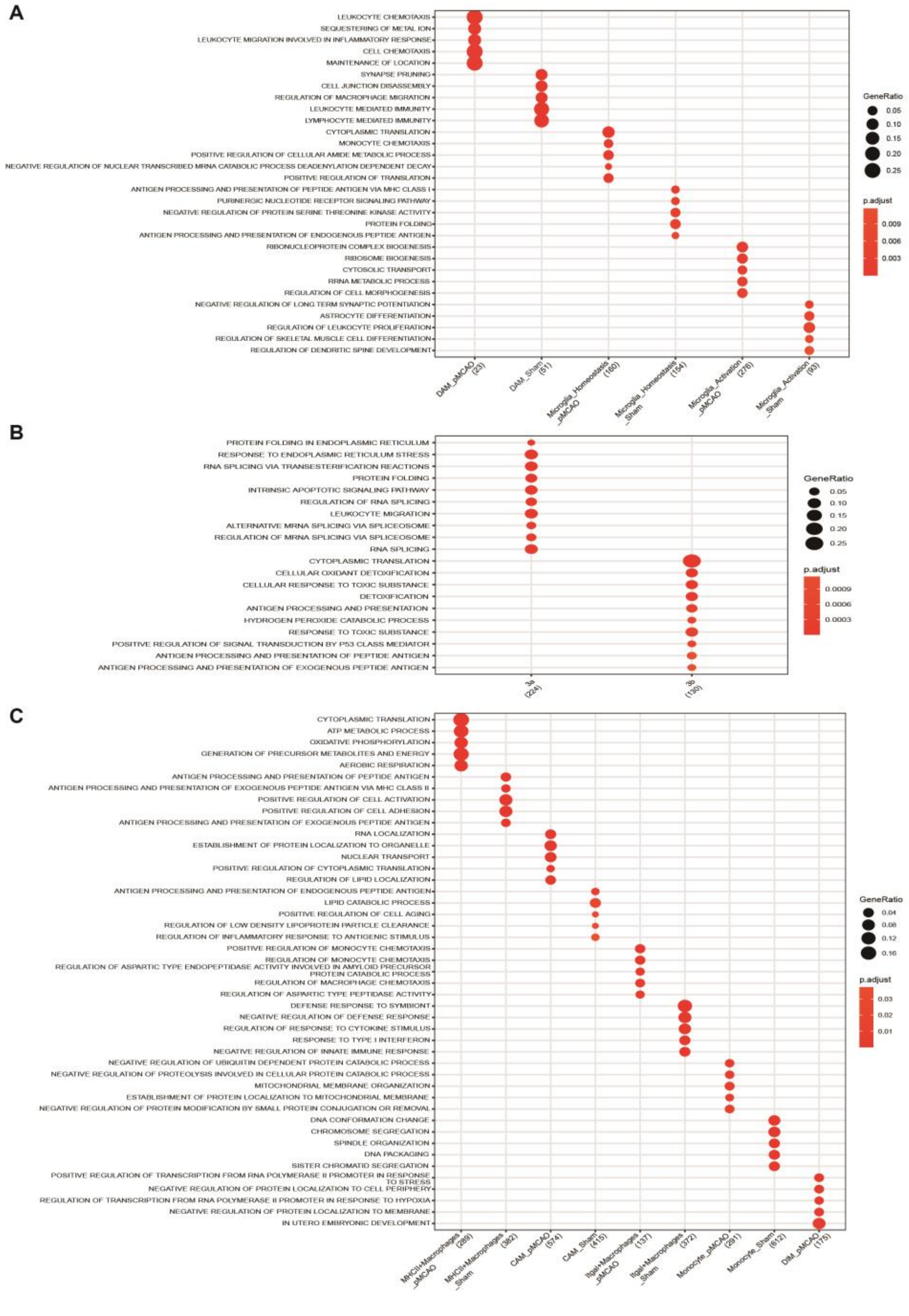

Fig. S12. GO function analysis of monocyte-macrophages and microglia.

(A) GO functional analysis of MCAO versus Sham controls was performed on selected subsets of microglia in the data set.

(B) GO function analysis in the 3a, and 3b clusters selected for this dataset.

(C) GO functional analysis of MCAO versus Sham controls was performed on selected subsets of mononuclear-macrophage in the data set.

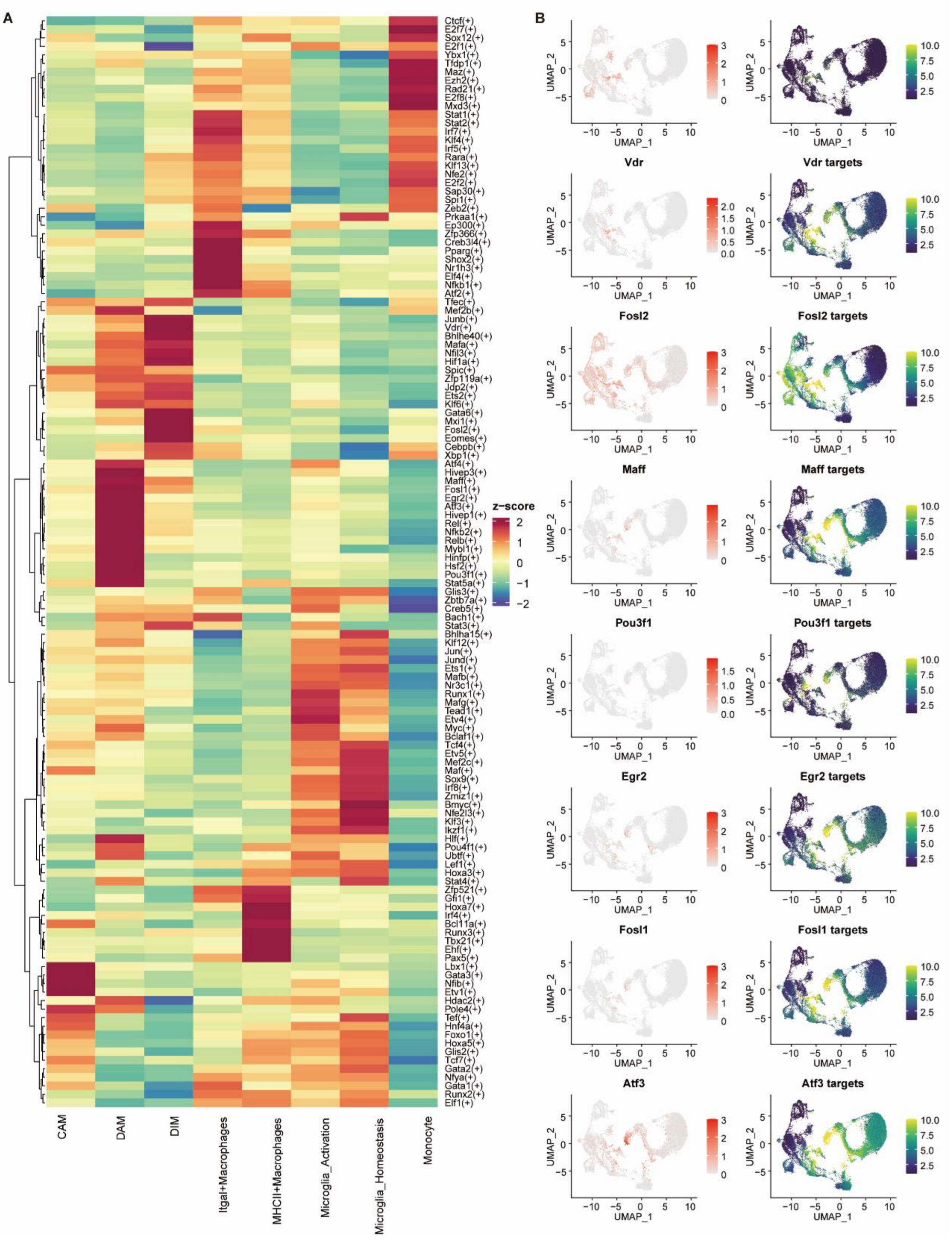

Fig. S13. Transcription factor regulatory network of monocytes/macrophages and microglia.

(A) Heatmap of transcription factors recognized by monocyte-derived macrophages and microglia.

(B) UMAP plot of transcription factor and target gene expression.

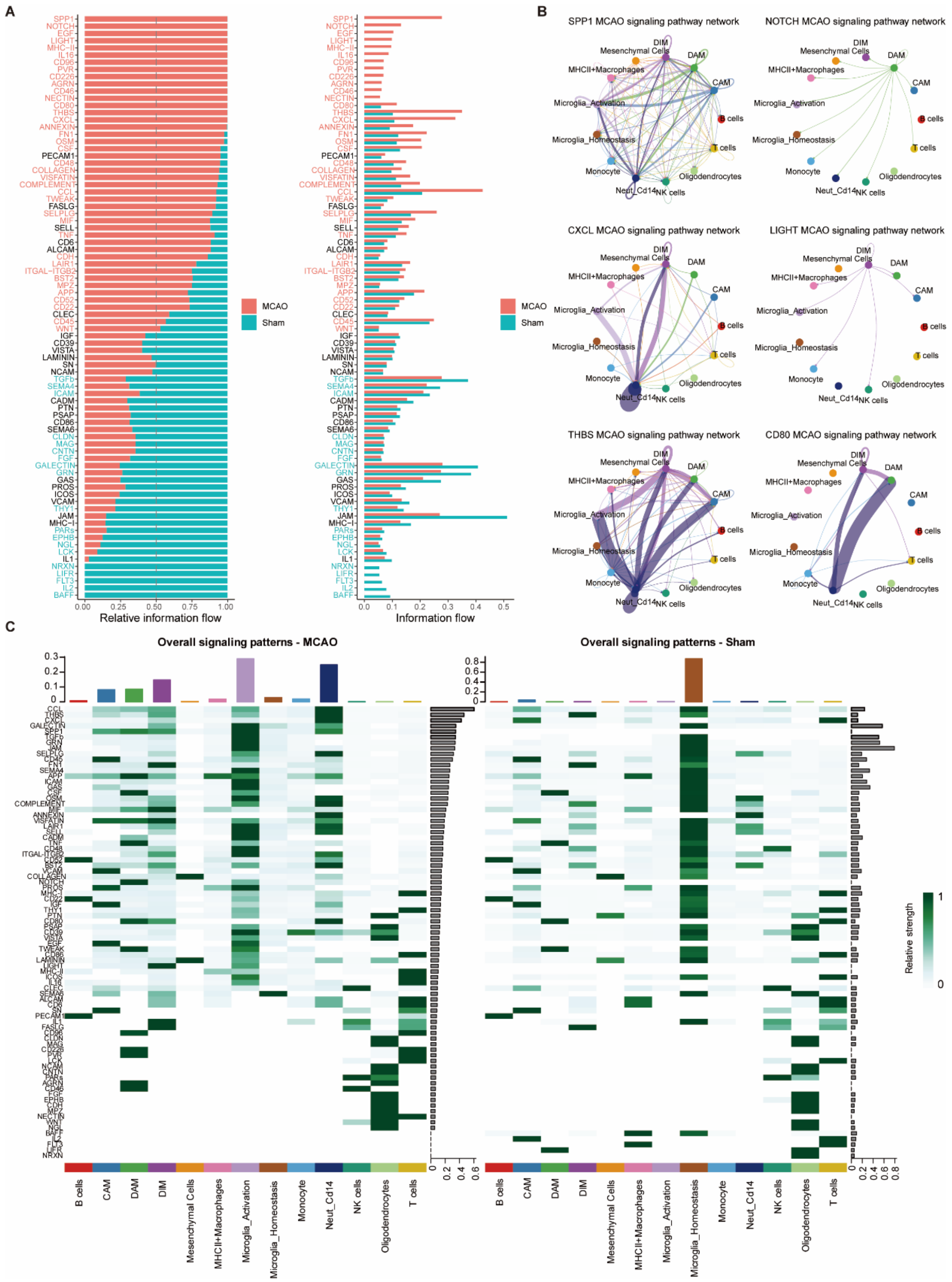

Fig. S14. Analysis of intercellular communication among different cerebral immune cell types.

(A) Analysis of differential intercellular communication in the brain between MCAO and sham groups, filtering cell populations with >10 cells.

(B) Communication signaling pathways associated with Neut_Cd14, DIM, and DAM.
(C) Comparison of cellular signaling patterns in the brain.

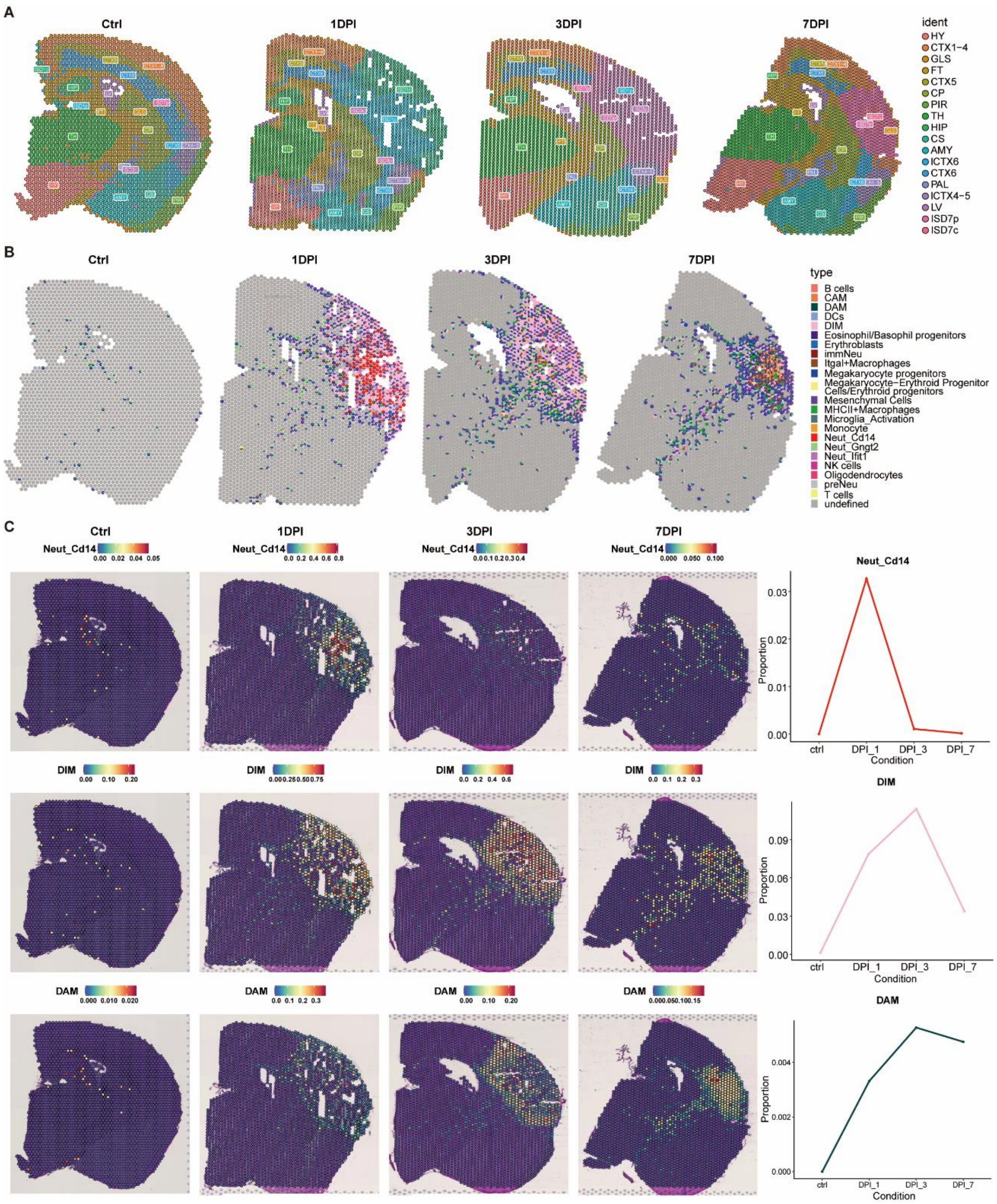

Fig. S15. Spatial plot of immune cells across different time points post-stroke.

(A) Spatial plot of color-coded by annotated brain regions (data from Zucha et al.).

(B) Spatial plot of deconvoluted cell type proportions per spot.

(C) Projection of Neut_Cd14, DIM, and DAM proportions and their changes across different states.

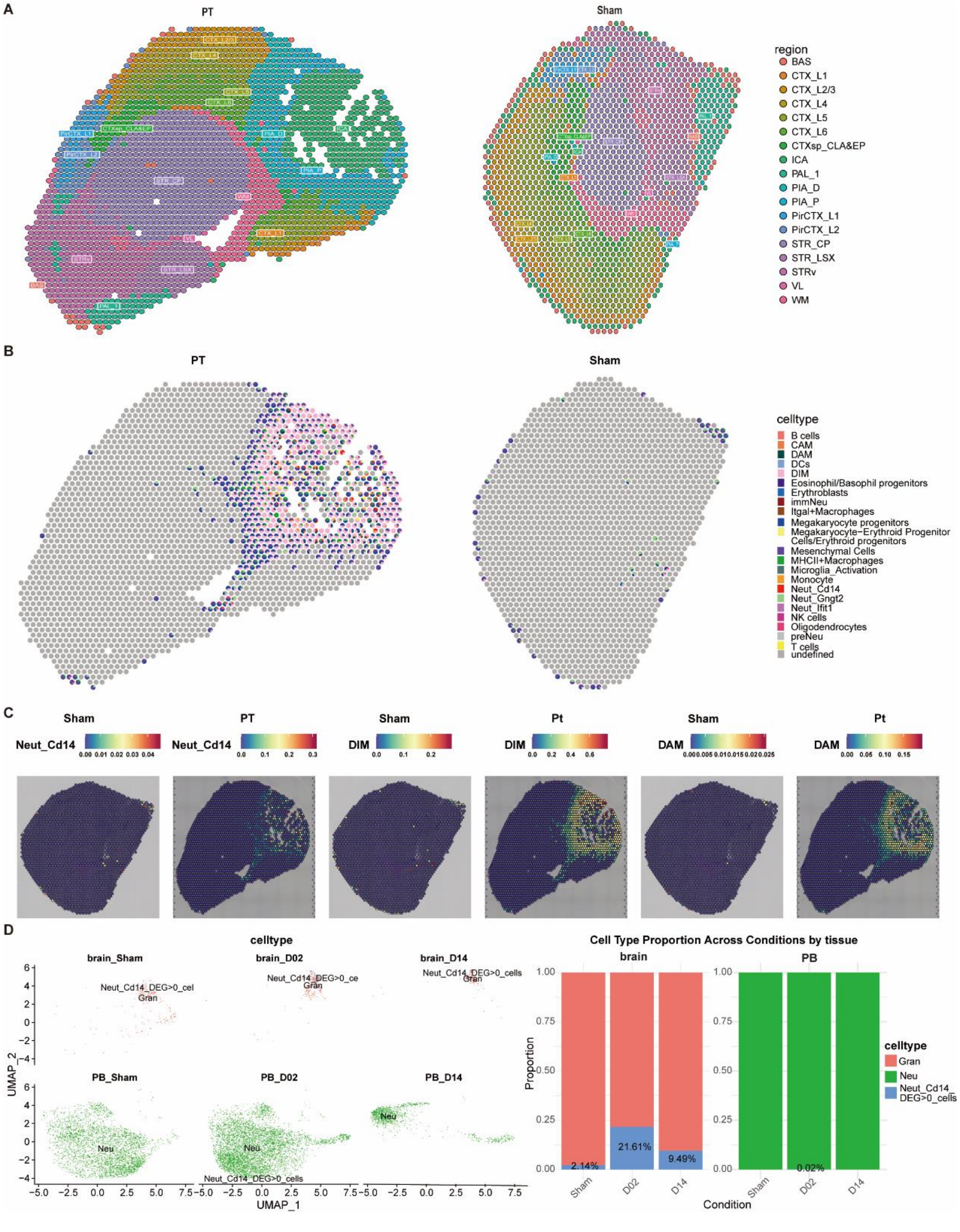

Fig. S16. Spatial and temporal analysis of cell types and immune cell proportions.

(A) Spatial plot of color-coded by annotated cell types (data from Han et al.).

(B) Spatial plot of deconvoluted cell type proportions per spot.

(C) Projection of Neut_Cd14, DIM, and DAM proportions and their changes across different states.

(D) Cell type distribution of neutrophils on Day 2 and Day 14 post-stroke from Garcia et al.(left), and changes in cell proportions across different tissues and states (right). With cells expressing all three Neut_Cd14 marker genes defined as Neut_Cd14_DEG>0_cells.

Table S1. Relative percentages of different cell types in each condition.

Major type	Cell type	Brain		CBM		FBM		PBMC	
		MCAO	Sham	MCAO	Sham	MCAO	Sham	MCAO	Sham
Stem cells	HSCs<br>(%)	1<br>(-*)	1<br>(-)	194<br>(1.7)	82<br>(1.0)	111<br>(1.3)	69<br>(0.7)	0<br>(-)	0<br>(-)
Lymphoid lineage	T cells<br>(%)	55<br>(0.6)	135<br>(1.0)	578<br>(5.1)	340<br>(4.0)	531<br>(6.0)	369<br>(3.8)	5376<br>(47.6)	3648<br>(31.5)
	B cells<br>(%)	125<br>(1.4)	68<br>(0.5)	2809<br>(24.6)	2799<br>(33.2)	2045<br>(23.2)	3822<br>(39.6)	1237<br>(10.9)	5009<br>(43.2)
	NK cells<br>(%)	10<br>(0.1)	45<br>(0.3)	179<br>(1.6)	108<br>(1.3)	113<br>(1.3)	81<br>(0.8)	399<br>(3.5)	686<br>(5.9)
Myeloid lineage	GMPs<br>(%)	23<br>(0.3)	5<br>(-)	152<br>(1.3)	55<br>(0.7)	87<br>(1.0)	49<br>(0.5)	10<br>(0.1)	0<br>(-)
	Neutrophils<br>(%)	1723<br>(19.6)	94<br>(0.7)	5057<br>(44.3)	4037<br>(47.9)	4203<br>(47.7)	4027<br>(41.7)	579<br>(5.1)	25<br>(0.2)
	Monocyte-Macrophages<br>(%)	1682<br>(19.2)	1054<br>(8.1)	1124<br>(9.9)	632<br>(7.5)	867<br>(9.8)	576<br>(6.0)	2491<br>(22.1)	496<br>(4.3)
	DCs<br>(%)	15<br>(0.2)	7<br>(-)	328<br>(2.9)	117<br>(1.4)	229<br>(2.6)	152<br>(1.6)	48<br>(0.4)	25<br>(0.2)
Erythroid and megakaryocytic lineage	Megakaryocyte-Erythroid Progenitor Cells/Erythroid progenitors<br>(%)	1<br>(-)	0<br>(-)	328<br>(2.9)	40<br>(0.5)	212<br>(2.4)	119<br>(1.2)	0<br>(-)	0<br>(-)
	Erythroblasts<br>(%)	2<br>(-)	2<br>(-)	294<br>(2.6)	61<br>(0.7)	287<br>(3.3)	93<br>(1.0)	0<br>(-)	0<br>(-)
	Erythroid cells<br>(%)	2<br>(-)	3<br>(-)	81<br>(0.7)	82<br>(1.0)	13<br>(0.1)	213<br>(2.2)	674<br>(6.0)	1273<br>(11.0)
	Megakaryocyte progenitors<br>(%)	0<br>(-)	1<br>(-)	0<br>(-)	0<br>(-)	0<br>(-)	0<br>(-)	456<br>(4.0)	351<br>(3.0)
Glial cells	Microglia<br>(%)	5018<br>(57.2)	11573<br>(88.7)	8<br>(-)	4<br>(-)	0<br>(-)	0<br>(-)	0<br>(-)	0<br>(-)
	Oligodendrocytes<br>(%)	13<br>(0.1)	23<br>(0.2)	0<br>(-)	0<br>(-)	0<br>(-)	0<br>(-)	0<br>(-)	0<br>(-)
Others	Eosinophil/Basophil progenitors<br>(%)	10<br>(0.1)	3<br>(-)	235<br>(2.1)	65<br>(0.8)	116<br>(1.3)	84<br>(0.9)	27<br>(0.2)	75<br>(0.6)
	Mesenchymal Cells<br>(%)	97<br>(1.1)	50<br>(0.4)	49<br>(0.4)	14<br>(0.2)	1<br>(-)	0<br>(-)	0<br>(-)	0<br>(-)
	Sum	8777	13064	11416	8436	8815	9654	11297	11588

* Cell subsets with cell number <10 were not counted in the percentage statistics and are shown as -.

Table S2. Relative percentages of different cell types for each condition for mononuclear-macrophages and microglia.

Celltype	Brain		CBM		FBM		PBMC	
	MCAO	Sham	MCAO	Sham	MCAO	Sham	MCAO	Sham
CAM (%)	694 (10.4)	719 (5.7)	29 (2.5)	5 (-)	6 (-)	2 (-)	17 (0.7)	1 (-)
DAM (%)	710 (10.6)	26 (0.2)	8 (-*)	1 (-)	3 (-)	1 (-)	3 (-)	2 (-)
DIM (%)	766 (11.5)	46 (0.4)	25 (2.1)	1 (-)	8 (-)	5 (-)	19 (0.8)	0 (-)
Itgal+Macrophages (%)	29 (0.4)	28 (0.2)	201 (17.1)	71 (10.9)	111 (12.9)	69 (11.7)	332 (13.4)	236 (48.8)
MHCII+Macrophages (%)	169 (2.5)	248 (2.0)	118 (10.1)	37 (5.7)	87 (10.1)	24 (4.1)	411 (16.6)	21 (4.3)
Microglia_Activation (%)	3798 (56.9)	215 (1.7)	14 (1.2)	5 (-)	8 (-)	3 (-)	8 (-)	0 (-)
Microglia_Homeostasis (%)	372 (5.6)	11205 (88.9)	7 (-)	7 (-)	8 (-)	3 (-)	9 (-)	9 (-)
Monocyte (%)	132 (2.0)	111 (0.9)	786 (67.0)	545 (83.5)	665 (77.1)	495 (84.2)	1691 (68.5)	227 (46.9)
Sum	6670	12598	1188	672	896	602	2490	496

* Cell subsets with cell number <10 were not counted in the percentage statistics and are shown as -.

Table S3. List of qRT-PCR primers used in this study.

Gene (mouse)	Primer sequence 5'-3'
Cd14	Forward Primer CACAATTCACTGCGGGATGC
	Reverse Primer AGCGAGTTTAGCTGACTGGG
Gapdh	Forward Primer CACTGAGCAAGAGAGGCCCTAT
	Reverse Primer GCAGCGAACTTTATTGATGGTATT
